# Facile Synthesis of the Naturally Cytotoxic Triterpenoid Saponin Patrinia-Glycoside B-II and Its Conformer

**DOI:** 10.3390/molecules181215193

**Published:** 2013-12-10

**Authors:** Li Ren, Yong-Xiang Liu, Dan Lv, Mao-Cai Yan, Han Nie, Yang Liu, Mao-Sheng Cheng

**Affiliations:** Key Laboratory of Structure-Based Drug Design and Discovery of Ministry of Education, Shenyang Pharmaceutical University, Shenyang 110016, China; E-Mails: rlsmile@163.com (L.R.); yongxiang.liu@syphu.edu.cn (Y.-X.L.); lvdanyezi@163.com (D.L.); yanmaocai@126.com (M.-C.Y.); niehan@gmail.com (H.N.)

**Keywords:** oleanolic acid saponin, stepwise glycosylation, tumor cytotoxicity, chair conformational fluctuation

## Abstract

The first chemical synthesis of the natural triterpenoid saponin Patrinia-glycoside B-II, namely oleanolic acid 3-*O*-α-l-rhamnopyranosyl-(1→2)-[β-d-gluco-pyranosyl-(1→3)]-α-l-arabinopyranoside, has been accomplished in a linear 11-step sequence 11 with 9.4% overall yield. The abnormal ^1^*C*_4_ conformation of the arabinose residue was found to occur *via* conformational fluctuation during preparation of the intermediates. Molecular mechanism and quantum chemistry calculations showed that Patrinia-glycoside B-II and its conformer **1** cannot interconvert under normal conditions. Preliminary structure-activity relationships studies indicated that the ^4^*C*_1_ chair conformation of the arabinose residue in the unique α-l-rhamnopyranosyl-(1→2)-α-l-arabinopyranosyl disaccharide moiety is one of the chief positive factors responsible for its cytotoxic activity against tumors.

## 1. Introduction

Triterpenoid saponins from terrestrial plants possess enormous structural diversity and varied biological activities [1,2,3]. Notably, some oleanane-type triterpenoid saponins bearing the α-l-rhamnopyranosyl-(1→2)-α-l-arabinopyranosyl disaccharide moiety at the C3-OH of the aglycone often present significant antitumor activity [4,5,6,7,8]. Previous research has identified this unique disaccharide moiety as a characteristic sugar sequence that can strongly induce the antitumor activity in some oleanane-type glycosides such as β-hederin [9,10].

**Figure 1:**
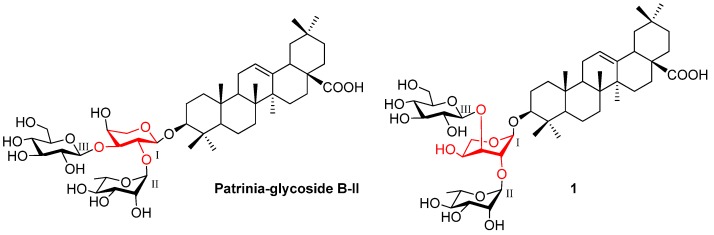
The structures of PB-II and its conformer **1**.

Patrinia-glycoside B-II (PB-II, Figure 1), a typical triterpenoid saponin isolated from the seeds of *Patrinia scabiosaefolia Fischer* [11], contains the unique oligosaccharide substructure and also displays prominent inhibitory activity against many tumor cell lines [7]. To investigate the structure-activity relationships (SARs) of this type of saponin, we have developed a convenient method for the chemical preparation of Patrinia-glycoside B-II.

## 2. Results and Discussion

### 2.1. Synthesis

The target compound consists of a trisaccharide moiety and oleanolic acid as the aglycone. With regard to the trisaccharide moiety, there is no neighboring participating group but rather a rhamnose residue connected to the C2-OH of arabinose. Because of this, the standard strategy of preparing a trisaccharide donor followed by coupling with the aglycone is not appropriate, as it would result in a mixture of α- and β-anomers. Accordingly, a stepwise glycosylation strategy was adopted in this work, as this strategy generally affords the 1,2-*trans*-glycoside linkage exclusively. Based on our experience, we chose isopropylidene, acetyl, and benzoyl groups as the temporary protecting groups for the hydroxyl groups of different sugars, and a benzyl group for the carboxylic acid of the oleanolic acid. In addition, perbenzoylated or peracetylated glycosyl trichloroacetimidates (**SD-1** to **SD-5**) were used as sugar donors, which were readily prepared from L-arabinose, L-rhamnose and D-glucose according to the reported methods [12,13].

**Scheme 1:**
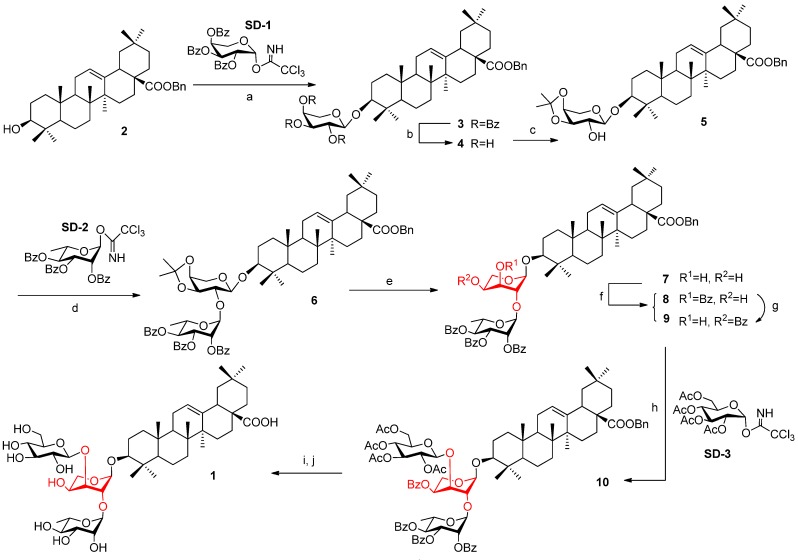
Synthesis of compound **1**.

As shown in Scheme 1, the benzyl ester of oleanolic acid **2** [9] was glycosylated with perbenzoylated arabinosyl trichloroacetimidate **SD-1** under trimethylsilyl trifluoromethanesulfonate (TMSOTf) catalysis to give compound **3** in excellent yield. Debenzoylation of compound **3** in a MeOH solution of NaOMe afforded saponin **4** without affecting the benzyl ester at C-28 of the aglycone. Selective protection of the 3^I^-OH and 4^I^-OH groups was successfully carried out using 2,2-dimethoxypropane (Me_2_C(OMe)_2_) to furnish product **5**. Then, the coupling of compound **5** with perbenzoylated rhamnosyl trichloroacetimidate **SD-2** under the same glycosylation conditions generated compound **6** in 79% yield. The isopropylidene moiety of compound **6** was removed with *para*-toluenesulfonic acid (*p*-TsOH), and the 3^I^-OH and 4^I^-OH were deprotected to afford compound **7**. However, products **8** and **9** were generated (in a ratio of 3:7) when we attempted to selectively protect the 4^I^-OH using Bu_2_SnO/BzCl [14]. This interesting phenomenon inspired us to check the structure of the former product **7**. Based on the ^1^H-NMR spectrum of compound **7**, the *J*_1'-2' _value of the arabinose residue was adjusted to 1.3 Hz, which is much smaller than the normal value of the α-arabinosyl conformation (usually no less than 5.0 Hz). Moreover, the chemical shift of the anomeric carbon (*δ* 109.3) is larger than that of the natural product (*δ* 104.8).

*Reagents and conditions*: (**a**) TMSOTf, DCM, 4Å MS, r.t., 97%; (**b**) NaOMe, DCM-MeOH (1:2, v/v), r.t., 96%; (**c**) Me_2_C(OMe)_2_, *p*-TsOH, DCM, r.t., 89%; (**d**) TMSOTf, DCM, 4Å MS, r.t., 79%; (**e**) *p*-TsOH, DCM-MeOH(1:2, v/v), r.t., 98%; (**f**) Bu_2_SnO, toluene, reflux; then BzCl, r.t., 94%; (**g**) BF_3_·Et_2_O, DCM, r.t.; (**h**) BF_3_·Et_2_O, DCM, 0 °C, 78% (for 2 steps); (**i**) 10% Pd-C, H_2_, EtOAc, reflux; (**j**) NaOMe, DCM-MeOH (1:2, v/v), rt, 45% (for 2 steps).

Hence, we realized that the ^1^*C*_4_ chair conformer of the arabinose residue was formed when compound **7** was produced. Based on our knowledge, the aglycone and perbenzoylated rhamnose (connected to the 1^I^-OH and 2^I^-OH of the arabinose residue, respectively) increase the steric bulk, resulting in the chair inversion of arabinose from the naturally stable ^4^*C*_1_ conformation to the ^1^*C*_4_ conformation. As expected, the 4^I^-OH of compound **7** is on the equatorial bond, which is favorable for generating the thermodynamically more stable compound **9** as the major species. Similar regioselectivity in 3-OH and 4-OH of arabinose has been reported previously [10,15,16,17,18], with the reduced activity of 3-OH rationalized by the presence of a bulky group at 2-OH. The polarities of compounds **8** and **9** were so similar that it was difficult to separate them by silica gel column chromatography. Fortunately, we found that compound **8** could be slowly converted into compound **9** in the presence of boron trifluoride ethyl etherate (BF_3_∙Et_2_O), *via* a benzoyl group migration [19]. Compound **9** was then reacted with peracetylated glucosyl donor **SD-3** under the promotion of BF_3_∙Et_2_O at 0 °C to give trisaccharide compound **10** in 78% yield. Finally, facile removal of the benzyl group through catalytic hydrogenation followed by debenzoylation and deacetylation in one pot yielded the conformer **1**. Interestingly, this novel saponin displayed a very robust structure; the ^1^*C*_4_ conformation of arabinose did not convert to the ^4^*C*_1_ form even under reflux conditions at 150 °C for 24 h.

**Scheme 2:**
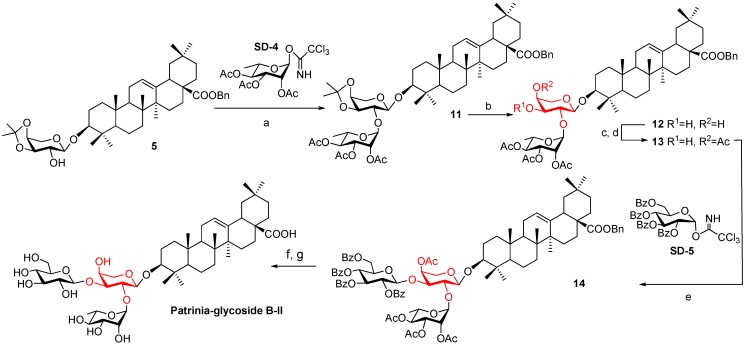
Synthesis of Patrinia-glycoside B-II.

Considering the negative influence of the bulky group at 2^I^-OH on the arabinose residue, we changed the perbenzoylated rhamnosyl trichloroacetimidate **SD-2** sugar donor to the much smaller **SD-4**. This time, compound **5** was glycosylated with **SD-4** at room temperature to give disaccharide saponin **11** in 73% yield. The isopropylidene group of compound **11** was removed by *p*-TsOH to generate the key intermediate **12**. Based on the ^1^H-NMR spectrum of compound **12**, the chemical shift (*δ* 4.73) and coupling constant (*J*_1'__-2'_ 5.0 Hz) of the anomeric 1^I^-H atom suggested that the arabinose ring maintained the normal ^4^*C*_1_ conformation. Next, compound **12** was converted into a cyclic ortho ester and was then cleaved in 50% HOAc to furnish product **13** with the 3^I^-OH on the equatorial bond. Compound **13** was able to couple with a bulkier sugar donor, such as perbenzoylated glucosyl trichloroacetimidate **SD-5**, under the promotion of BF_3_∙Et_2_O at a low temperature. Finally, removal of the benzyl group and all the acyl groups generated the target saponin (Scheme 2). In general, the natural Patrinia-glycoside B-II was synthesized in 11 linear steps from oleanolic acid with a 9.4% overall yield. The ^1^H-NMR and ^13^C-NMR spectra are consistent with those of the natural product. Therefore, Patrinia-glycoside B-II and its conformer were both prepared.

*Reagents and conditions*: (**a**) TMSOTf, DCM, 4Å MS, r.t., 73%; (**b**) *p*-TsOH, DCM-MeOH, r.t., 89%; (**c**) CH_3_C(OEt)_3_, *p*-TsOH, DCM, r.t.; (**d**) HOAc-H_2_O (50%), r.t., 76% (for 2 steps); (**e**) BF_3_·Et_2_O, DCM, 4Å MS, −78 °C; (**f**) Pd-C, H_2_, EtOAc, reflux; (**g**) NaOMe, DCM-MeOH, r.t., 23% (for 3 steps).

### 2.2. Computational Calculations

To determine the conditions in which the two compounds could interconvert, the Sybyl 6.9 software package (Tripos, st. Louis, MO, USA) was used to analyze structural conformations of compound **1**. The structure of compound **1** was built with the Sketcher module, and the energy was minimized by Powell’s method using the Tripos force field. Gasteiger-Hückel charges were used for electrostatic field computations. The minimization was terminated after 1000 steps at a maximum value of the gradient at 0.05 kcal/mol^−1^∙Å^−1^. The structure was then subjected to a simulated annealing, in which it was heated from 200 K to 1,000 K and annealed slowly to 200 K. The results showed that the ^1^*C*_4_ and ^4^*C*_1_ conformations are stable and unable to interconvert at 1000 K. In addition to the two-chair conformations, some boat conformations with irregular conformation are also stable conformations. Next, a 300 K to 500 K simulated annealing was used to study the structural conformations: besides the original ^1^*C*_4_ conformation, the boat with irregular conformations also has low energy, but the ^4^*C*_1_ conformation could not be found. This suggests that the arabinosyl conformation of compound **1** might not be able to convert to a ^4^*C*_1_ conformation at 500 K.

**Figure 2:**
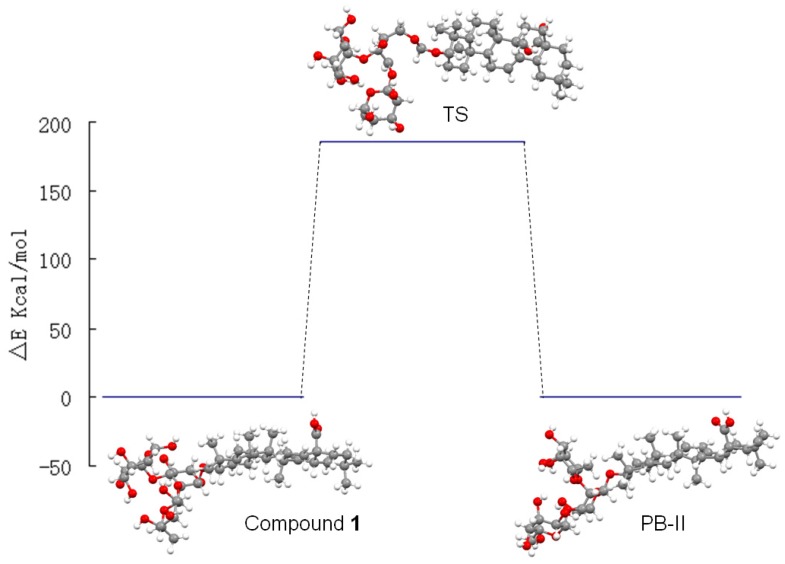
The energy gap between different conformers.

**Figure 3:**
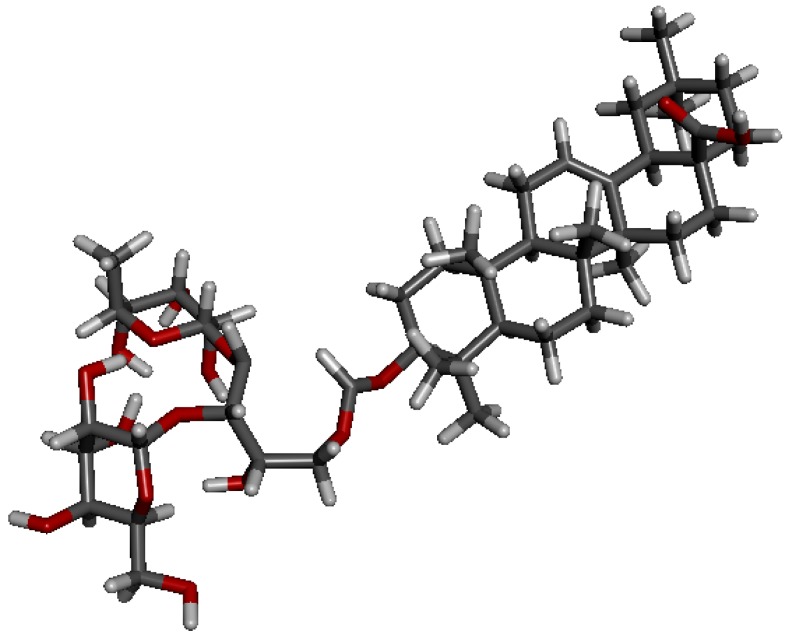
The transition state conformation.

Further tautomerism studies were carried out using the Amsterdam Density Functional (ADF) program [20]. The geometry optimizations for compound **1**, Patrinia-glycoside B-II and the transition state were performed in a vacuum. The total energy values were obtained from the optimization output and are listed in Table 1. From the results, it can be observed that both compound **1** and Patrinia-glycoside B-II are energetically stable. Although the energy gap between them is only 0.314 kcal/mol, interconversion would be difficult given the large energy barrier of 185.79 kcal/mol (Figure 2). Further, the C_1_-C_2_ chemical bond needs to be broken and created, as shown by the transition state (Figure 3). Therefore, both molecular mechanism and quantum chemistry calculations showed that compound **1** and Patrinia-glycoside B-II cannot interconvert under normal conditions.

**Table 1:** The energy of compound **1**, Patrinia-glycoside B-II and Transition State.

	Compound 1	Patrinia-Glycoside B-II	Transition State
Energy(Kcal/mol)	−19,986.949	−19,987.263	−19,801.469

### 2.3. Biological Evaluation

A preliminary *in vitro* pharmacology assay was then performed to evaluate the cytotoxicity of these saponins against eight human cancer cell lines using a standard MTT method, in which 5-fluorouracil (5-FU) was chosen as the positive control. As shown in Table 2, Patrinia-glycoside B-II showed effective inhibitory activity against all of those tumor cell lines at micromolar concentrations. Whereas compound **1** did not exhibit significantly cytotoxicity against any tumor cell lines compared to Patrinia-glycoside B-II and 5-FU. This result indicated that the ^4^*C*_1_ chair conformation of the arabinose residue in the unique α-l-rhamnopyranosyl-(1→2)-α-l-arabinopyranosyl disaccharide moiety is one of the chief positive factors responsible for its cytotoxic activity against tumors.

**Table 2:** The IC_50_^a^^,b^ (µmol·L) of 5-FU, PB-II and compound **1** on various tumor cell lines.

Compd	HeLa	HepG2	HT1080	A549	A375-S2	K562	HL60	U937
PB-II	5.4 ± 0.2	4.2 ± 0.8	18.0 ± 0.5	27.9 ± 0.8	15.8 ± 0.1	6.2 ± 0.6	6.6 ± 0.3	5.5 ± 0.3
Compound **1**	>100	>100	>100	>100	>100	>100	>100	>100
5-FU	>50	>50	15.2	>50	25.31.6	36.4 ± 3.1	9.6 ± 0.9	18.2 ± 2.0

^a^ Concentration inhibiting fifty percent of cell growth for 48 h exposure period of tested samples. Data represent mean values ± standard deviation for independent experiments; ^b^ IC_50_s more than 50 µM are not shown their exact values.

## 3. Experimental

### 3.1. General

Commercial reagents were used without further purification unless specialized. Solvents were dried and redistilled prior to use in the usual way. Boiling range of petroleum ether was 60–90 °C. Analytical TLC was performed with silica gel HF_254_. Preparation column chromatography was performed with silica gel H. Melting points were detected with a Büchi B-540 Melting Point apparatus. Optical rotations were measured at the sodium D-line at room temperature with a Perkin-Elmer 241MC polarimeter. ^1^H and ^13^C-NMR spectra were recorded on an Avance AV 600 MHz spectrometer using Me_4_Si as the internal standard. The high resolution mass spectrometry (HRMS) measurements were performed using a high resolution Fourier Transform ion cyclotron resonance (FT-ICR) instrument (Bruker, Germany) operated in electrospray ionization mode.

### 3.2. Benzyl Oleanolate 3-O-3,4-O-isopropylidene-α-l-arabinopyranoside (**5**)

The benzyl ester of oleanolic acid **2** (1.1 g, 2.01 mmol), trichloroacetimidate **SD-1** (1.27 g, 2.10 mmol) and powdered 4Å molecular sieve (MS 4Å, 500 mg) were mixed in dry dichloromethane (DCM, 25 mL) and allowed to stir at r.t. for 20 min. A dry DCM solution (2 mL) of TMSOTf (0.20 mL, 0.10 mmol) was then added dropwise. The mixture was stirred for about 1 h until the starting materials were completely consumed to form a new compound. The mixture was neutralized with triethylamine (Et_3_N, 0.2 mL, 1.43 mmol) and filtered through a pad of Celite. The filtrate was concentrated and purified by silica gel column chromatography (8:1, petroleum ether–ethyl acetate (EtOAc)) to afford compound **3** (1.87 g, 97%) as a white foam. To a solution of compound **3** (1.5 g, 1.50 mmol) in DCM–MeOH (1:2, v/v, 40 mL) was added a solution of NaOMe in MeOH (1.00 mol/L, 1.70 mL). The mixture was allowed to stir at r.t. for 2 h and neutralized with Dowex H^+^ resin and then filtered. The filtrate was concentrated and the residue was subjected to a silica gel column chromatography (EtOAc) to give saponin **4** (991 mg, 96%) as a white amorphous solid. To a solution of compound **4** (679 mg, 1.00 mmol) in dry acetone (10 mL) was added Me_2_C(OMe)_2_ (0.31 mL, 2.50 mmol) and *p*-TsOH (17.20 mg, 0.10 mmol). The mixture was stirred for 4 h before Et_3_N (0.20 mL, 1.43 mmol) was added. The solution was concentrated and the residue was purified by silica gel column chromatography (6:1, petroleum ether–EtOAc) to afford a white foam **5** (634 mg, 89%) with *R*_f_ 0.45 (4:1, petroleum ether-EtOAc). 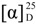 +45.0 (*c* 1.60, CHCl_3_); Mp 76.8–79.2 °C; ^1^H-NMR (600 MHz, CDCl_3_): *δ* 7.34 (m, 5H, Ar-H), 5.28 (t, *J =* 3.0 Hz, 1H, H-12), 5.07 (dd, *J =* 18.7, 12.6 Hz, 2H, PhC*H*_2_), 4.22–4.17 (m, 3H, H-1', H-4', H-5'-1), 4.06 (dd, *J =* 7.8, 6.1 Hz, 1H, H-3'), 3.75 (dd, *J =* 13.9, 3.5 Hz, 1H, H-5'-2), 3.63 (d, *J =* 7.8 Hz, 1H, H-2'), 3.12 (dd, *J =* 11.5, 4.6 Hz, 1H, H-3), 2.91 (dd, *J =* 13.8, 3.3 Hz, 1H, H-18), 2.30 (br s, 1H, O*H*), 1.54, 1.36 (s each, 3H each, O-(C*H*_3_)_2_C-O), 1.11, 0.98, 0.92, 0.89, 0.88, 0.82, 0.60 (s each, 3H each, 7 × Me); ^13^C-NMR (150 MHz, CDCl_3_): *δ* 177.4, 143.6, 136.3, 128.3, 127.9, 127.8, 122.4, 110.0, 104.3, 88.9, 78.1, 74.2, 73.2, 65.9, 63.0, 55.4, 47.5, 46.6, 45.8, 41.6, 41.3, 39.2, 39.0, 38.4, 36.6, 33.8, 33.1, 32.6, 32.3, 30.6, 28.2, 28.0, 27.5, 26.0, 25.8, 23.6, 23.3, 22.9, 18.1, 16.8, 16.6, 15.2; HRMS: calcd for C_38_H_59_O_7_ (M-Bn)^−^: 627.4261; Found: *m/z* 627.4255.

### 3.3. Benzyl Oleanolate 3-O-2,3,4-tri-O-benzoyl-α-l-rhamnopyranosyl-(1→2)-3,4-O-isopropylidene-α-l-arabinopyranoside (**6**)

A mixture of compound **5** (560 mg, 0.78 mmol), trichloroacetimidate **SD-2** (630 mg, 1.00 mmol) and powdered MS 4Å (300 mg) in dry DCM (10 mL) was stirred at r.t. for 20 min. A dry DCM solution of TMSOTf (0.10 mL, 0.05 mmol) was then added dropwise and the mixture was stirred for 2 h, followed by the addition of Et_3_N (0.20 mL, 1.43 mmol) and filtration. The filtrate was concentrated and subjected to a silica gel chromatography (8:1, petroleum ether–EtOAc) to furnish disaccharide saponin **6** (730 mg, 79%) as a white foam with *R*_f_ 0.60 (4:1, petroleum ether–EtOAc). 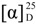 +96.7 (*c* 2.58, CHCl_3_); Mp 122.5–124.2 °C; ^1^H-NMR (600 MHz, CDCl_3_): *δ* 8.12–7.21 (m, 20H, Ar-H), 5.87 (dd, *J =* 10.2, 3.3 Hz, 1H, H-3''), 5.76 (brs, 1H, H-1''), 5.65 (m, 2H, H-2'', H-4''), 5.30 (brs, H-12), 5.07 (dd, *J =* 22.4, 12.6 Hz, 2H, PhC*H*_2_), 4.53 (m, 1H, H-5''), 4.47 (d, *J =* 7.8 Hz, 1H, H-1'), 4.25 (m, 2H, H-3', H-4'), 4.17 (m, 1H, H-5'-1), 3.90 (d, *J =* 7.8 Hz, 1H, H-2'), 3.79 (m, 1H, H-5'-2), 3.17 (dd, *J =* 11.3, 4.1 Hz, 1H, H-3), 2.92 (m, 1H, H-18), 1.55, 1.35 (s each, 3H each, O-(C*H*_3_)_2_C-O), 1.34 (d, *J =* 6.1 Hz, 3H, H-6''), 1.14, 0.95, 0.93, 0.92, 0.90, 0.89, 0.64 (s each, 3H each, 7 × Me); ^13^C-NMR (150 MHz, CDCl_3_): *δ* 177.4, 165.7, 165.5, 165.4, 143.7, 136.4, 133.3, 133.2, 132.9, 130.0, 129.7, 129.6, 129.5, 129.4, 129.3, 128.5, 128.4, 128.3, 128.2, 127.9, 122.5, 110.4, 103.5, 95.4, 89.4, 79.4, 75.5, 73.6, 72.2, 70.8, 70.1, 66.7, 66.1, 62.9, 56.0, 47.6, 46.7, 45.8, 41.7, 41.4, 39.3, 39.2, 38.7, 36.7, 33.8, 33.1, 32.7, 32.4, 30.7, 28.2, 27.8, 27.6, 26.1, 25.9, 23.6, 23.4, 23.0, 18.1, 17.5, 16.9, 16.7, 15.3; HRMS (ESI): Calcd for C_65_H_81_O_14_ (M-Bn)^−^: 1085.5626; Found: *m/z* 1085.5619.

### 3.4. Benzyl Oleanolate 3-O-2,3,4-tri-O-benzoyl-α-l-rhamnopyranosyl-(1→2)-α-l-arabinopyranoside (**7**)

Compound **6** (705 mg, 0.60 mmol) was dissolved in a solution of DCM–MeOH (1:2, v/v, 40 mL) and then *p*-TsOH (78 mg, 0.45 mmol) was added. The solution was stirred at r.t. for 3 h when Et_3_N (0.40 mL, 2.86 mmol) was added and the mixture was concentrated and the residue was purified by silica gel column chromatography (2:1, petroleum ether–EtOAc) to give compound **7** (666 mg, 98%) as a white amorphous solid with *R*_f_ 0.36 (2:1, petroleum ether–EtOAc). 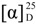 +77.3 (*c* 2.27, CHCl_3_); Mp 143.7–147.2 °C; ^1^H-NMR (600 MHz, CDCl_3_): *δ* 8.10–7.23 (m, 20H, Ar-H), 5.82 (dd, *J =* 10.2, 3.1 Hz, 1H, H-3"), 5.69 (m, 2H, H-2'', H-4''), 5.34 (s, 1H, H-1''), 5.29 (br s, 1H, H-12), 5.07 (dd, *J =* 18.7, 12.6 Hz, 2H, PhCH_2_), 4.81 (d, *J =* 3.0 Hz, 1H, H-1'), 4.34 (m, 1H, H-5''), 4.11–3.98 (m, 3H, H-2', H-4', OH), 3.82 (m, 1H, H-5'-1), 3.67 (m, 1H, H-5'-2), 3.45 (d, *J =* 7.9 Hz, 1H, H-3'), 3.18 (dd, *J =* 11.0, 3.2 Hz, 1H, H-3), 2.91 (m, 1H, H-18), 2.52 (br s, 1H, OH), 1.34 (d, *J =* 6.1 Hz, 3H, H-6''), 1.12, 1.05, 0.92, 0.89, 0.88, 0.84, 0.61 (s each, 3H each, 7 × Me); ^13^C-NMR (150 MHz, CDCl_3_): *δ* 177.4, 165.7, 165.5, 143.6, 136.4, 133.5, 133.3, 133.1, 129.9, 129.7, 129.6, 129.2, 129.2, 129.1, 128.5, 128.4, 128.2, 127.9, 127.8, 122.4, 102.0, 98.2, 90.3, 76.2, 71.5, 70.7, 70.7, 69.7, 67.3, 65.9, 65.5, 61.1, 55.4, 47.6, 46.7, 45.8, 41.6, 41.3, 39.2, 39.1, 38.5, 36.7, 33.8, 33.1, 32.6, 32.3, 30.6, 28.1, 27.6, 25.8, 25.7, 23.6, 23.4, 23.0, 18.2, 17.5, 16.8, 16.4, 15.3; HRMS: calcd for C_62_H_77_O_14_ (M-Bn)^−^: 1045.5313; found: *m/z* 1045.5307.

### 3.5. Benzyl Oleanolate 3-O-2,3,4-tri-O-benzoyl-α-l-rhamnopyranosyl-(1→2)-3-O- benzoyl-a-l-arabinopyranoside (**8**) and benzyl oleanolate 3-O-2,3,4-tri- O-benzoyl-α-l-rhamnopyranosyl-(1→2)-4-O-benzoyl-α-l-arabinopyranoside (**9**)

Compound **7** (300 mg, 0.26 mmol) and Bu_2_SnO (72 mg, 0.29 mmol) were dissolved in dry toluene (6 mL) and heated to reflux. After stirring for 1 h, the solution was cooled to r.t. followed by the dropwise addition of a toluene solution of BzCl (10% V/V, 370 μL). 4 h later, the mixture was quenched by Et_3_N, then concentrated and purified by silica gel column chromatography (4:1, petroleum ether–EtOAc) to give the mixture of **8** and **9** (309 mg, 94%).

Compound **8**: *R*_f _0.37 (4:1, petroleum ether–EtOAc); Mp 128.6–134.6 °C; ^1^H-NMR (600 MHz, CDCl_3_): *δ* 8.10–7.21 (m, 25H, Ar-H), 5.80 (dd, *J =* 10.1, 2.4 Hz, 1H, H-3''), 5.65 (m, 2H, H-2'', H-4''), 5.36 (s, 1H, H-1''), 5.32 (dd, *J =* 5.8, 2.8 Hz, 1H, H-3'), 5.29 (br s, 1H, H-12), 5.09 (dd, *J =* 33.0, 12.6 Hz, 2H, PhCH_2_), 4.76 (d, *J =* 3.8 Hz, 1H, H-1'), 4.41 (m, 1H, H-5''), 4.33 (m, 1H, H-2'), 4.29 (m, 1H, H-4'), 4.08 (m, 1H, H-5'-1), 3.71 (br d, *J =* 9.6 Hz, 1H, H-5-2), 3.18 (dd, *J =* 11.5, 3.8 Hz, 1H, H-3), 2.91 (dd, *J =* 13.9, 3.6 Hz, 1H, H-18), 1.33 (d, *J =* 6.0 Hz, 3H, H-6''), 1.11, 1.03, 0.92, 0.90, 0.87, 0.70, 0.60 (s, 3H each, 7 × Me); HRMS: calcd for C_69_H_81_O_15_ (M-Bn)^−^: 1149.5576; found: *m/z* 1149.5569.

Compound **9**: *R*_f_ 0.37 (4:1, petroleum ether–EtOAc); Mp 147.0–152.8 °C; ^1^H-NMR (600 MHz, CDCl_3_): *δ* 8.12–7.25 (m, 25H, Ar-H), 5.89 (dd, *J =* 10.2, 3.3 Hz, 1H, H-3''), 5.78 (m, 1H, H-2''), 5.71 (t, *J =* 10.0 Hz, 1H, H-4''), 5.47 (s, 1H, H-1''), 5.39 (m, 1H, H-4'), 5.30 (br s, 1H, H-12), 5.07 (dd, *J =* 33.1, 12.5 Hz, 2H, PhCH_2_), 4.82 (d, *J =* 4.1 Hz, 1H, H-1'), 4.45 (m, 1H, H-5''), 4.26 (m, 1H, H-3'), 4.15 (dd, *J =* 12.1, 6.9 Hz, 1H, H-5'-1), 4.06 (dd, *J =* 4.6, 5.6 Hz, 1H, H-2'), 3.83 (dd, *J =* 12.1, 3.4 Hz, 1H, H-5'-2), 3.21 (dd, *J =* 11.7, 4.3 Hz, 1H, H-3), 2.91 (dd, *J =* 13.8, 3.8 Hz, 1H, H-18), 1.37 (d, *J =* 6.2 Hz, 3H, H-6''), 1.13, 1.11, 0.92, 0.90, 0.90, 0.90, 0.62 (s, 3H each, 7 × Me). HRMS: calcd for C_69_H_81_O_15_ (M-Bn)^−^: 1149.5576; found: *m/z* 1149.5569.

### 3.6. Benzyl Oleanolate 3-O-2,3,4-tri-O-benzoyl-α-l-rhamnopyranosyl-(1→2)- [2,3,4,6-tetra-O-acetyl-β-D-glucopyranosyl-(1→3)]-3-O-benzoyl-α-l-arabinopyranoside (**10**)

To a solution of the mixture of **8** and **9** (300 mg, 0.24 mmol) in dry DCM (8 mL) was added BF_3_-Et_2_O (5% V/V, 0.80 mL) and stirred at r.t. for 6 h when TLC showed complete conversion of the **8**. Then, a dry DCM solution of trichloroacetimidate **SD-3** (5.80 mL, 0.2 mol/L) was added dropwise at 0 °C. The mixture was allowed to stir for 1 h followed by the addition of Et_3_N (1 mL, 7.15 mmol) and filtration. The filtrate was concentrated and subjected to a silica gel chromatography (3:1, petroleum ether-EtOAc) to furnish trisaccharide saponin **10** (197 mg, 78% in 2 steps) with *R*_f_ 0.23 (3:1, petroleum ether–EtOAc). 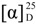 +32.6 (*c* 1.75, CHCl_3_); ^1^H-NMR (600 MHz, CDCl_3_): *δ* 8.12–7.25 (m, 25H, Ar-H); 5.77 (dd, *J =* 10.3, 3.3 Hz, 1H, H-2''), 5.73 (m, 1H, H-3''), 5.69 (t, *J =* 10.0 Hz, 1H, H-4''), 5.39 (s, 1H, H-1''), 5.31 (s, 1H, H-1'), 5.30 (br s, 1H, H-12), 5.28 (dd, *J =* 9.9, 9.6 Hz, 1H, H-3'''), 5.22 (d, *J =* 3.0 Hz, 1H, H-3'), 5.13 (dd, *J =* 9.4, 10.4 Hz, 1H, H-4'''), 5.11-5.03 (m, 3H, H-2''', PhCH_2_), 4.74 (d, *J =* 8.2 Hz, 1H, H-1'''), 4.48 (m, 1H, H-4'), 4.39 (br s, 1H, H-2'), 4.29 (dd, *J =* 12.4, 4.1 Hz, 1H, H-6'''-1), 4.23 (dd, *J =* 10.0, 5.9 Hz, 1H, H-5'-1), 4.19-4.17 (m, 2H, H-5'', H-6'''-2), 3.81–3.78 (m, 2H, H-5'-2, H-5'''), 3.16 (dd, *J =* 11.3, 4.4 Hz, 1H, H-3), 2.91 (dd, *J =* 13.7, 3.4 Hz, 1H, H-18), 2.07, 2.01, 1.99, 1.96 (s, 3H each, 4 × CH_3_CO), 1.35 (d, *J =* 6.1 Hz, 3H, H-6''), 1.13, 0.95, 0.92, 0.90, 0.90, 0.76, 0.61 (s, 3H each, 7 × Me). HRMS: calcd for C_82_H_97_O_24_ (M-Bn)^−^: 1465.6370; found: *m/z* 1465.6364.

### 3.7. Oleanolic Acid 3-O-α-l-rhamnopyranosyl-(1→2)-[β-D-glucopyranosyl-(1→3)]-α-l-arabino-pyranoside (**1**)

A suspension of compound **10** (180 mg, 0.12 mmol) and 10% Pd/C (80 mg) in EtOAc (30 mL) was stirred under reflux and bubbled with H_2_ (25 mL/min) for 2 h. Pd/C was removed through a pad of Celite and the filtrate was concentrated. The sticky residue was dissolved in dry DCM–MeOH (1:2, v/v, 9 mL), to which a freshly prepared NaOMe solution in MeOH (1.0 mol/L, 1.50 mL) was added. The solution was stirred at r.t. for 4 h and then neutralized with Dowex H^+^ resin to pH 7 and filtered. The filtrate was concentrated and subjected to a silica gel column chromatography (7:3:1, CHCl_3_–MeOH–H_2_O) to give a white powder **1** (46 mg, 45%, for 2 steps) with *R*_f_ 0.25 (7:3:1, CHCl_3_–MeOH–H_2_O). 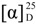 −38.5 (*c* 0.52, MeOH); Mp 210.4–213.8 °C;^ 1^H-NMR (600 MHz, pyridine-*d*_5_): *δ* 5.87 (s, 1H, H-1''), 5.51 (s, 1H, H-1'), 5.48 (br s, 1H, H-12), 4.98 (d, *J =* 7.8 Hz, 1H, H-1'''), 4.92–4.90 (dd, *J =* 1.8 Hz, 1H, H-2'), 4.75–4.72 (m, 1H, H-4''), 4.70–4.68 (m, 1H, H-3'), 4.57–4.55 (m, 1H, H-2''), 4.53–4.49 (m, 3H, H-5'-1, H-3'', H-6'''-1), 4.42-4.20 (m, 6H, H-4', H-6'''-2, H-5'', H-5'-2, H-3''', H-4'''), 4.06 (m, 1H, H-2'''), 3.94 (m, 1H, H-5'''), 3.29 (dd, *J =* 13.8, 3.8 Hz, 1H, H-18), 3.18 (dd, *J =* 11.7 Hz, 4.3, 1H, H-3), 1.69 (d, *J =* 6.2 Hz, 3H, H-6''), 1.27, 1.11, 1.00, 0.98, 0.94, 0.90, 0.80 (s, 3H each, 7 × Me); ^13^C-NMR (150 MHz, pyridine-*d*_5_): *δ* 180.5, 145.1, 122.9, 109.6, 105.4, 101.5, 88.6, 87.7, 82.3, 78.9, 78.8, 78.4, 75.4, 74.3, 72.9, 72.8, 71.9, 70.6, 70.4, 63.0, 56.0, 48.3, 47.0, 46.8, 42.5, 42.3, 40.0, 39.4, 38.9, 37.3, 34.6, 33.6, 33.6, 33.5, 31.3, 28.7, 28.6, 26.5, 26.5, 24.1, 24.1, 24.0, 18.9, 18.9, 17.7, 17.3, 15.8; HRMS (*m/z*): calcd for C_47_H_76_NaO_16_ (M+Na)^+^: 919.5026; found: 919.5030.

### 3.8. Benzyl Oleanolate 3-O-2,3,4-tri-O-acetyl-α-l-rhamnopyranosyl-(1→2)-3,4-O- isopropylidene-α-l-arabinopyranoside (**11**)

A suspension of compound **5** (450 mg, 0.63 mmol), trichloroacetimidate **SD-4** (1.25 g, 1.50 mmol) and powdered MS 4Å (300 mg) in dry DCM (15 mL) was stirred at r.t. for 20 min and then TMSOTf (0.10 mL, 0.05 mmol) was added. The solution was stirred for 2 h before the reaction was quenched with Et_3_N (1 mL, 7.15 mmol). The mixture was filtered and the filtrate was concentrated and subjected to a silica gel chromatography (8:1, petroleum ether–EtOAc) to furnish compound **11** (450 mg, 73%) as a white foam with *R*_f_ 0.55 (4:1, petroleum ether–EtOAc). 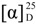 +6.8 (*c* 0.62, CHCl_3_); ^1^H-NMR (600 MHz, CDCl_3_): *δ* 7.35–7.34 (m, 5H, Ar-H), 5.34 (brs, 1H, H-1''), 5.33-5.32 (m, 2H, H-2'', H-3''), 5.28 (brs, 1H, H-12), 5.08 (dd, *J =* 20.1, 12.6 Hz, 2H, PhCH_2_), 4.36 (d, *J =* 7.2 Hz, 1H, H-1'), 4.21 (m,1H, H-3'), 4.20 (m, 1H, H-5'') 4.19 (m, 1H, H-4'), 4.17 (t, *J =* 7.2 Hz, 1H, H-2'), 3.77 (dd, *J =* 13.8, 5.4 Hz, 1H, H-5'-1), 3.75 (dd, *J =* 13.2, 3.6 Hz, 1H, H-5'-2), 3.09 (dd, *J =* 11.3, 4.2 Hz, 1H, H-3), 2.91 (dd, *J =* 10.2, 2.8 Hz, 1H, H-18), 2.15, 2.02, 1.97 (s, 3H each, 3 × CH_3_CO), 1.53, 1.34 (s, 3H each, O-(CH_3_)_2_C-O), 1.20 (d, *J =* 6.0 Hz, 3H, H-6''), 1.12, 1.03, 0.92, 0.89, 0.89, 0.82, 0.61 (s each, 3H each, 7 × Me); ^13^C-NMR (150 MHz, CDCl_3_): *δ* 177.6, 170.4, 170.2, 143.9, 136.6, 128.6, 128.2, 122.7, 110.6, 103.4, 95.4, 89.3, 79.4, 75.3, 73.6, 71.4, 69.8, 69.3, 66.4, 66.1, 62.8, 56.1, 47.8, 46.9, 46.1, 41.6, 39.3, 38.9, 36.9, 34.1, 33.3, 32.9, 32.6, 30.9, 29.9, 28.3, 27.9, 27.8, 26.3, 26.1, 23.8, 23.6, 21.2, 21.0, 20.9, 18.4, 17.6, 17.1, 16.6, 15.6; HRMS (*m/z*): calcd for C_58_H_84_NaO_13_ (M+Na)^+^: 1013.5610; found: 1013.5615.

### 3.9. Benzyl Oleanolate 3-O-2,3,4-tri-O-acetyl-α-l-rhamnopyranosyl-(1→2)-α-l- arabinopyranoside (12)

To a solution of compound **11** (300 mg, 0.30 mmol) in a solution of DCM-MeOH (1:2, v/v, 25 mL), was added *p*-TsOH (104 mg, 0.60 mmol) and the mixture was stirred at room temperature. When TLC (2:1, petroleum ether-EtOAc) showed the deprotection was complete, Et_3_N (0.5 mL, 3.58 mmol) was added to quench the reaction and the mixture was concentrated and purified by silica gel column chromatography (2:1, petroleum ether–EtOAc) to give compound **12** (254 mg, 89%) as a white amorphous solid with *R*_f_ 0.36 (2:1, petroleum ether–EtOAc). 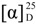 +41.0 (*c* 0.19, CHCl_3_); Mp 142.7–149.6 °C; ^1^H-NMR (CDCl_3_): *δ* 7.36–7.31 (m, 5H, Ar-H), 5.29–5.26 (m, 3H, H-3'', H-4'', H-12), 5.10–5.05 (m, 3H, H-2'', PhCH_2_), 5.03 (s, 1H, H-1''), 4.71 (d, *J =* 5.0 Hz, 1H, H-1'), 4.00 (m, 1H, H-5''), 3.93 (m, 1H, H-4'), 3.89-3.85 (m, 2H, H-2', H-3'), 3.74 (dd, *J =* 11.4, 4.2 Hz, 1H, H-5'-1), 3.61 (dd, *J =* 11.4, 4.2 Hz, 1H, H-5'-2), 3.13 (dd, *J =* 11.4, 4.2 Hz, 1H, H-3), 2.91 (dd, *J =* 13.8, 3.6 Hz, 1H, H-18), 2.15, 2.05, 2.00 (s, 3H each, 3 × CH_3_CO), 1.20 (d, *J =* 6.0 Hz, 3H, H-6''), 1.12, 0.97, 0.93, 0.90, 0.89, 0.80, 0.60 (s, 3H each, 7 × Me); ^13^C-NMR (CDCl_3_): *δ* 128.6, 128.2, 128.1, 122.6, 102.0, 98.4, 76.1, 71.1, 69.9, 69.1, 67.2, 66.2, 47.8, 46.9, 46.1, 41.9, 41.6, 39.5, 39.3, 38.7, 36.9, 33.3, 32.9, 32.6, 30.9, 28.3, 27.8, 26.1, 25.9, 23.9, 23.6, 23.3, 21.1, 21.0, 18.5, 17.6, 17.1, 16.6, 15.5; HRMS (*m/z*): calcd for C_55_H_80_NaO_13_ (M+Na)^+^: 973.5278; found: 973.5282.

### 3.10. Benzyl Oleanolate 3-O-2,3,4-tri-O-acetyl-α-l-rhamnopyranosyl-(1→2)-4-O- acetyl-α-l-arabino-pyranoside (**13**)

A mixture of compound **12** (200 mg, 0.21 mmol), CH_3_C(OEt)_3_ (0.50 mL, 4.02 mmol) and *p*-TsOH (25 mg, 0.15 mmol) in dry DCM (12 mL) was stirred at r.t. for 1 h before the reaction was quenched with Et_3_N (0.5 mL). The solution was concentrated and the residue was dissolved in 50% a.q. HOAc (10 mL) and stirred at r.t. for 2 h. Then the solvents were co-evaporated with toluene for complete removal of HOAc. The straw yellow residue was then subjected to a silica gel chromatography (2:1, petroleum ether–EtOAc) to give compound **13** (159 mg, 76%) as a white foam with *R*_f _0.27 (2:1, petroleum ether–EtOAc). 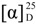 +91.0 (*c* 0.10, CHCl_3_); ^1^H-NMR (600 MHz, CDCl_3_): *δ* 7.34–7.27 (m, 5H, Ar-H), 5.32–5.31 (m, 3H, H-4', H-3'', H-2''), 5.13 (brs, 1H, H-1''), 5.11–5.04 (m, 4H, PhCH_2_, H-12, H-2'), 4.09 (d, *J =* 6.6 Hz, 1H, H-1'), 3.98–3.95 (m, 1H, H-4''), 3.94–3.89 (m, 1H, H-3'), 3.86–3.84 (m, 1H, H-5'-1), 3.64 (dd, *J =* 3. 6, 12.0 Hz, 1H, H-5'-2), 3.13 (dd, *J =* 4.2, 1.2 Hz, 1H, H-3), 2.91(dd, 1H, *J =* 3.6, 10.2 Hz, H-18), 2.15, 2.13, 2.05 (s, 3H each, 3 × CH_3_CO), 1.22 (d, *J =* 6.0 Hz, 3H, H-6''), 1.12, 1.00, 0.99, 0.93, 0.90, 0.81, 0.60 (s, 3H each, 7 × Me); ^13^C-NMR (150 MHz, CDCl_3_): *δ* 177.7, 170.87, 170.2, 143.9, 136.7, 129.1, 128.6, 128.2, 128.1, 122.7, 102.9, 98.2, 90.6, 77.4, 77.2, 77.0, 76.1, 71.2, 69.9, 69.1, 67.2, 66.2, 55.8, 47.8, 47.0, 46.1, 41.9, 41.6, 39.5, 39.3, 38.8, 36.9, 34.1, 33.3, 32.9, 32.6, 30.9, 28.3, 27.8, 26.1, 23.9, 23.6, 23.3, 21.3, 21.1, 21.0, 20.9, 19.4, 18.5, 17.6, 17.1, 16.6; HRMS (*m/z*): calcd for C_57_H_82_NaO_14_ (M+Na)^+^: 1015.5480; found: 1015.5484.

### 3.11. Oleanolic Acid 3-O-α-l-rhamnopyranosyl-(1→2)-[β-d-glucopyranosyl-(1→3)]-α-l-arabino-pyranoside (Patrinia-glycoside B-II)

To a solution of compound **13** (80 mg, 0.084 mmol) in dry DCM (8 mL) was added BF_3_-Et_2_O (0.016 mL, 0.13 mmol) and powdered MS 4Å (50 mg). The mixture was stirred at −78 °C for 15 min, then trichloroacetimidate **SD-5** (75.0 mg, 0.101 mmol) was added and the mixture was stirred for 1 h before Et_3_N (0.16 mL, 1.15 mmol) was added to quench the reaction. The mixture was then diluted with DCM (25 mL) and filtered. The filtrate was concentrated and purified by silica gel column chromatography (5:1, petroleum ether–EtOAc) to afford compound **14** (118 mg, 90%) as a white foam. A suspension of compound **14** (118 mg, 0.075 mmol) and 10% Pd/C (60 mg) in EtOAc (10 mL) was stirred under reflux and bubbled up with H_2_ (25 mL/min) for 5 h. Pd/C was removed through filtration and the filtrate was concentrated to dryness. The resulted residue was dissolved in a dry solution of DCM–MeOH (1:2, v/v, 24 mL), to which a freshly prepared NaOMe solution in MeOH (1.0 mol/L, 0.30 mL) was added. The mixture was stirred at r.t. for 2 h and then neutralized with Dowex H^+^ resin to pH 7 and filtered. The filtrate was concentrated and subjected to a silica gel column chromatography (7:3:1, CHCl_3_–MeOH–H_2_O) to give a pale yellow powder, which was recrystallized in MeOH to give the target saponin (20 mg, 23%) as a white powder *R*_f_ 0.23 (7:3:1, CHCl_3_–MeOH–H_2_O). Mp 260.2–262.3 °C; 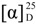 −27.5 (*c* 0.49, MeOH); ^1^H-NMR (600 MHz, pyridine-*d*_5_): *δ* 6.20 (br s, 1H, H-1''), 5.47 (br s, 1H, H-12), 5.13 (d, 1H, *J =* 7.8 Hz, H-1'''), 4.87 (d, *J =* 5.4 Hz, 1H, H-1'), 4.77 (br s, 1H, H-2''), 4.68 (d, 1H, *J =* 7.8 Hz, H-2'), 4.66–4.50 (m, 4H, H-5'', H-3'', H-4', H-6'''-1), 4.41–4.18 (m, 7H, H-5'-1, H-5'-2, H-6'''-2, H-3', H-3''', H-4''', H-4''), 3.97-3.94 (m, 2H, H-2''', H-5'''), 3.40 (d, *J =* 14.4 Hz, 1H, H-18), 3.18 (d, *J =* 10.8 Hz, 1H, H-3), 1.55 (d, *J =* 6.0 Hz, 3H, H-6''), 1.26, 1.18, 1.08, 0.97, 0.92, 0.87, 0.80 (s, 3H each, 7 × Me); ^13^C-NMR (150 MHz, pyridine-*d*_5_): *δ* 179.9, 144.5, 122.2, 104.5, 104.5, 101.7, 87.9, 82.0, 78.3, 78.0, 74.7, 74.5, 73.7, 72.3, 72.3, 71.2, 69.8, 67.9, 64.8, 62.3, 55.7, 47.8, 46.4, 46.2, 46.0, 41.9, 41.7, 39.5, 39.3, 38.7, 36.8, 34.0, 33.0, 33.0, 31.9, 30.7, 29.8, 29.4, 28.1, 27.8, 26.4, 28.1, 27.8, 26.4, 25.9, 23.5, 23.4, 18.4, 18.2, 17.1, 16.8, 15.3; HRMS (*m/z*): calcd for C_47_H_76_NaO_16 _(M+Na)^+^: 919.5026; found: 919.5030.

### 3.12. Computational Methods

All computational calculations were performed using the Sybyl software package and Amsterdam Density Functional (ADF) program. PBE functional and standard DZP basis set with large frozen core are used in geometry optimization and transition state search.

### 3.13. Cell Culture

HeLa human cervical cancer cells; HepG2 human hepatoma cells; HT1080 human fibrosarcoma cells; A549 human lung cancer cells; A375-S2 human melanoma cell; K562 human malignant myeloid cell; HL-60 human promyelocytic leukemia cell and U937 human lymphoma cell were obtained from the American Type Culture Collection (Rockville, MD, USA). The cancer cells were maintained in RPMI 1640 medium with 10% fetal bovine serum (FBS); 2% glutamine. Cultures were maintained in a humidified atmosphere incubator at 37 °C in 5% CO_2_.

### 3.14. Cell Viability Assay

The cell viability was evaluated using MTT assay. After diluting to 5 × 10^4^ cells mL^−^^1^ with the complete medium, 100 μL of the obtained cell suspension was added to each well of 96-well culture plates. The subsequent incubation was permitted at 37 °C, 5% CO_2_ atmosphere for 24 h before the cytotoxicity assessments. Tested samples at pre-set concentrations were added to each well, and then the cells were incubated for 48 h. The MTT solution (100 μL, 0.5 mg/mL) was added to each well, and the cells were incubated for another 4 h. The formazan crystals were dissolved in 150 μL of DMSO. Cell viability was assessed by measuring the absorbance at a 492 nm wavelength using an EMax Microplate Reader (Molecular Devices, Sunnyvale, CA, USA).

## 4. Conclusions

In summary, the natural cytotoxic triterpenoid saponin Patrinia-glycoside B-II and its novel conformer **1** were synthesized by a stepwise glycosylation strategy. The abnormal ^1^*C*_4_ conformation of the arabinose residue was found to occur *via* conformational fluctuation during preparation of the intermediates. Molecular mechanism and quantum chemistry calculations showed that compound **1** and Patrinia-glycoside B-II cannot interconvert under normal conditions. Preliminary structure-activity relationships studies indicated that the ^4^*C*_1_ chair conformation of the arabinose residue in the unique α-l-rhamnopyranosyl-(1→2)-α-l-arabinopyranosyl disaccharide moiety is one of the chief positive factors responsible for its cytotoxic activity against tumors.

## Supplementary Materials

Supplementary materials can be accessed at: http://www.mdpi.com/1420-3049/18/12/15193/s1.
