# Supplementary material for: Facile Synthesis of the Naturally Cytotoxic Triterpenoid Saponin Patrinia-Glycoside B-II and Its Conformer
Source: Molecules. 2013 Dec 10;18(12):15193–206. doi: 10.3390/molecules181215193 (PMC6270016; doi:10.3390/molecules181215193)

Supplementary Materials

Table S1. Optical rotation and melting point of Nature PB-II, Synthetic PB-II and Compound **1**.

	Nature PB-II	Synthetic PB-II	Compound 1
$[\alpha]_D^{25}$	−2.9 (*c* 0.29, Py)	−27.5 (*c* 0.49, MeOH)	−38.5 (*c* 0.52, MeOH)
Mp	259.0–262.0 °C	260.2–262.3 °C	210.4–213.8 °C

Table S2. ^{1}H-NMR data of Nature PB-II, Synthetic PB-II and Compound **1**.

Hydro No	Nature PB-II	Synthetic PB-II	Compound 1
Ara-H1	4.85 (d, *J* = 5.1 Hz)	4.87 (d, *J* = 5.4 Hz)	5.51 (s)
Rha-H1	6.11 (brs)	6.20 (brs)	5.87 (brs)
Glu-H1	5.08 (d, *J* = 7.7 Hz)	5.13(d, *J* = 7.8 Hz)	4.98 (d, *J* = 7.8 Hz)

Table S3. ^{13}C-NMR data of Nature PB-II, Synthetic PB-II and Compound **1**.

Carbon No	Nature product	Synthetic product	Compound 1
Ara			
C-1	104.8	104.1	109.6
C-2	74.8	74.5	88.6
C-3	82.2	82.0	78.4
C-4	68.1	67.9	82.3
C-5	64.9	64.8	70.6
Rha			
C-1	101.9	101.7	101.5
C-2	72.3	72.3	72.9
C-3	72.5	72.3	72.8
C-4	73.9	73.7	74.3
C-5	70.0	69.8	70.6
C-6	18.6	18.2	
Glu			
C-1	104.7	104.1	105.4
C-2	74.9	74.7	75.4
C-3	78.2	78.3	78.9
C-4	71.4	71.2	71.9
C-5	76.5	78.0	78.8
C-6	62.5	62.3	63.0

^{1}H-, ^{13}C- and 2D-NMR data for compounds 1, 6-9, 11-13, PB-II

Figure S1. Structure of compound **6**.

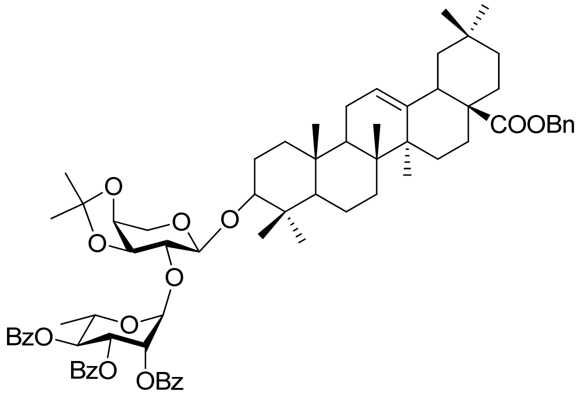

Figure S2. ^{1}H-NMR of compound **6**.

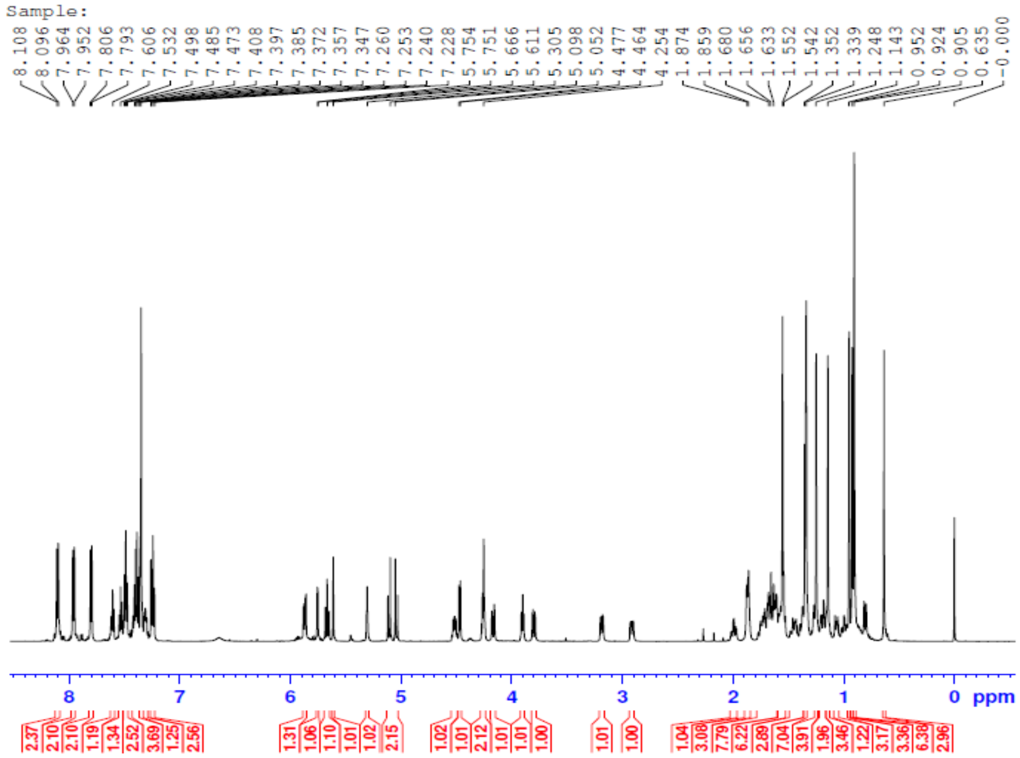

Figure S3. ^{13}C-NMR of compound **6**.

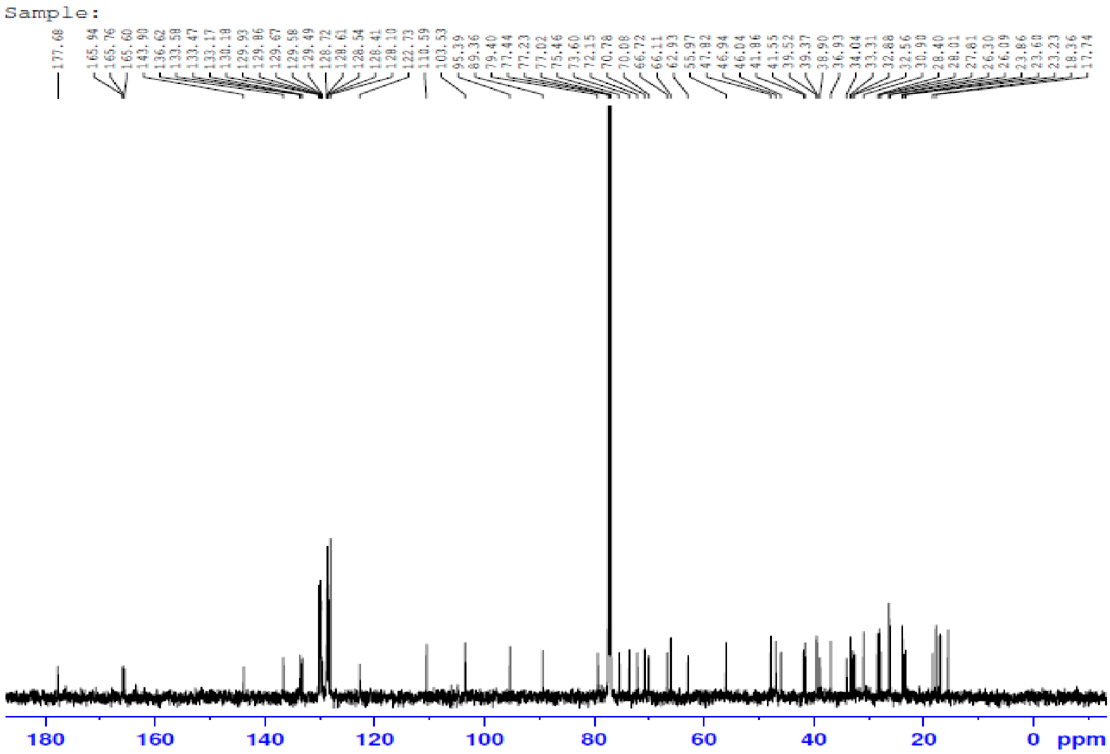

Figure S4. COSY of compound **6**.

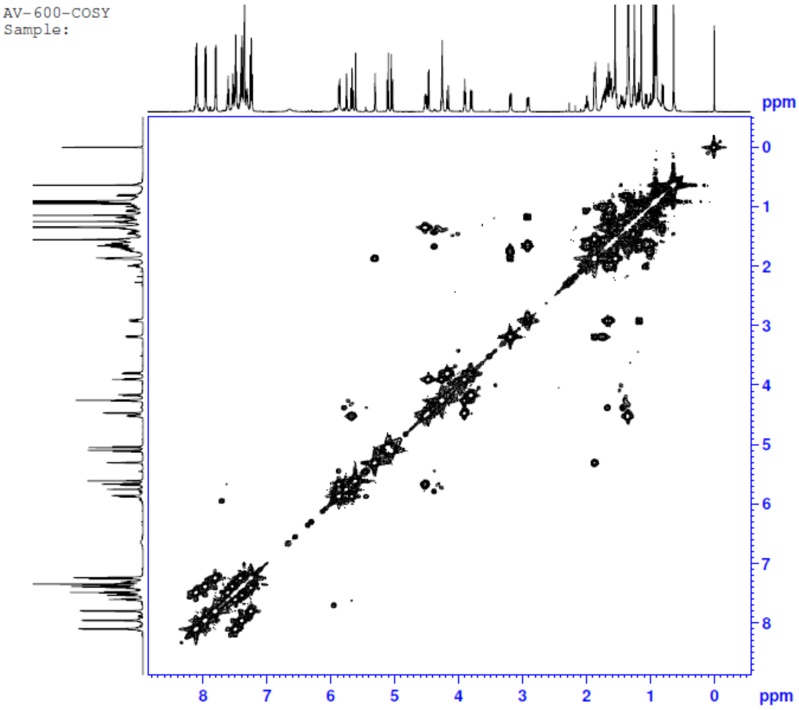

Figure S5. HSQC compound **6**.

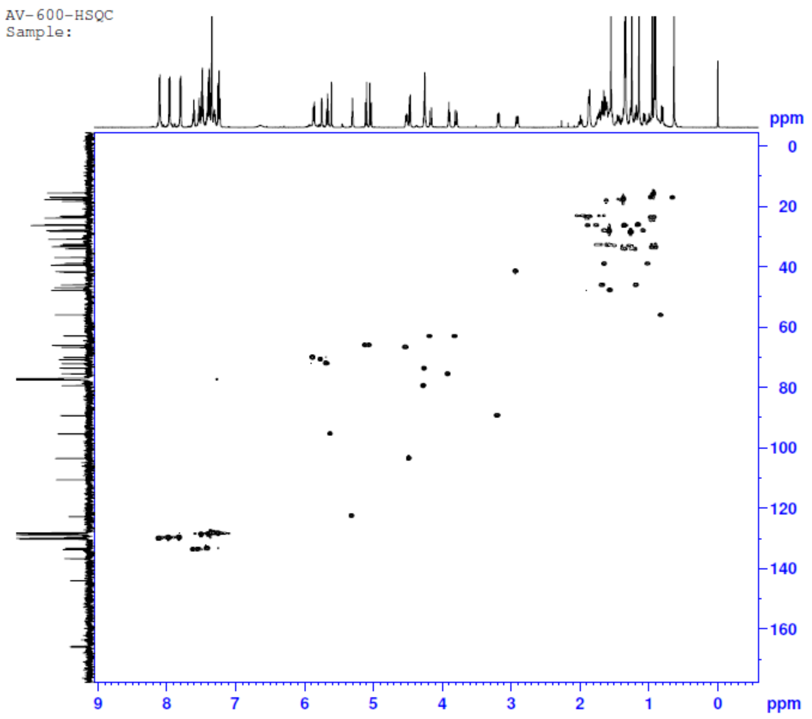

Figure S6. HMBC compound **6**.

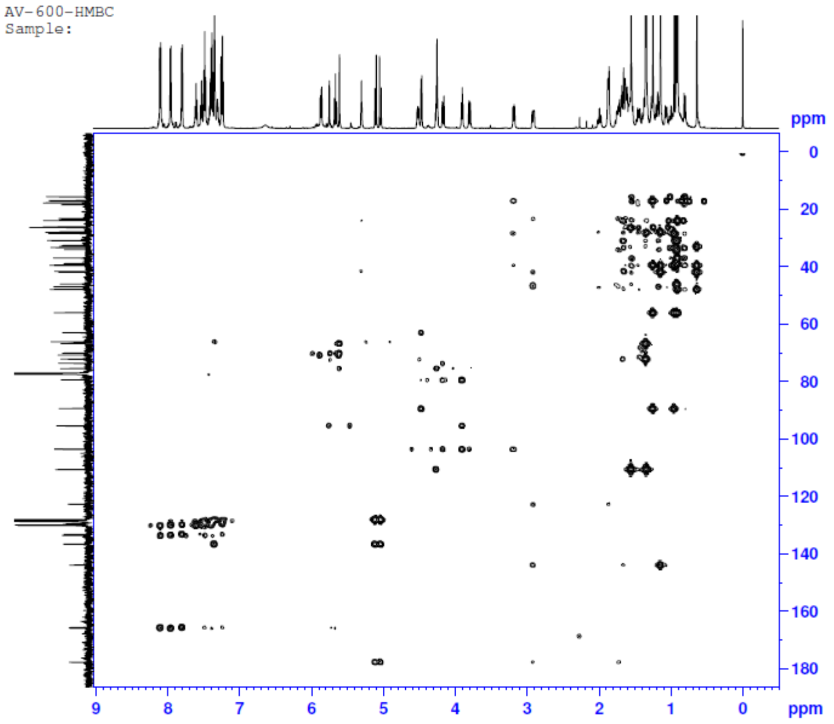

Figure S7. Structure of compound **7**.

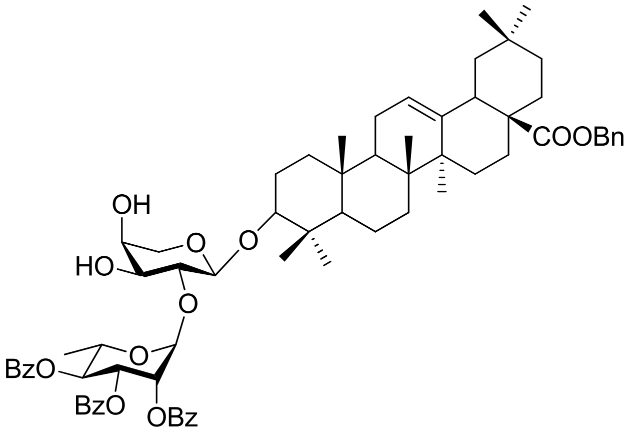

Figure S8. ^{1}H-NMR of compound **7**.

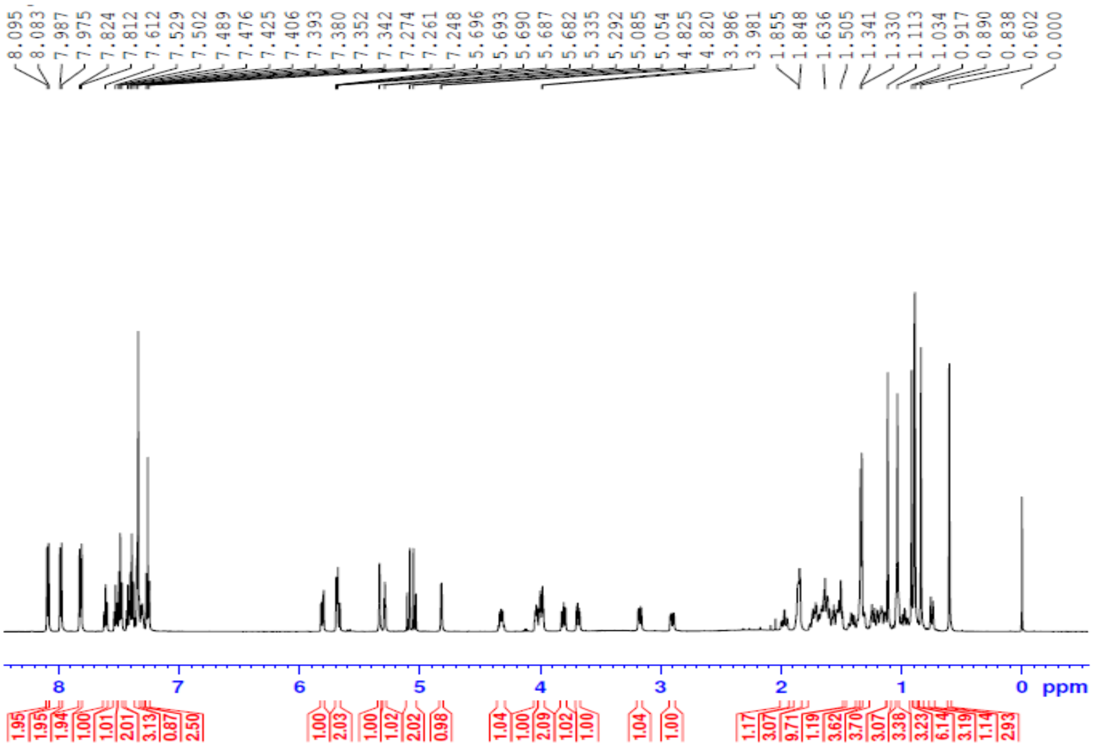

Figure S9. ^{13}C-NMR of compound **7**.

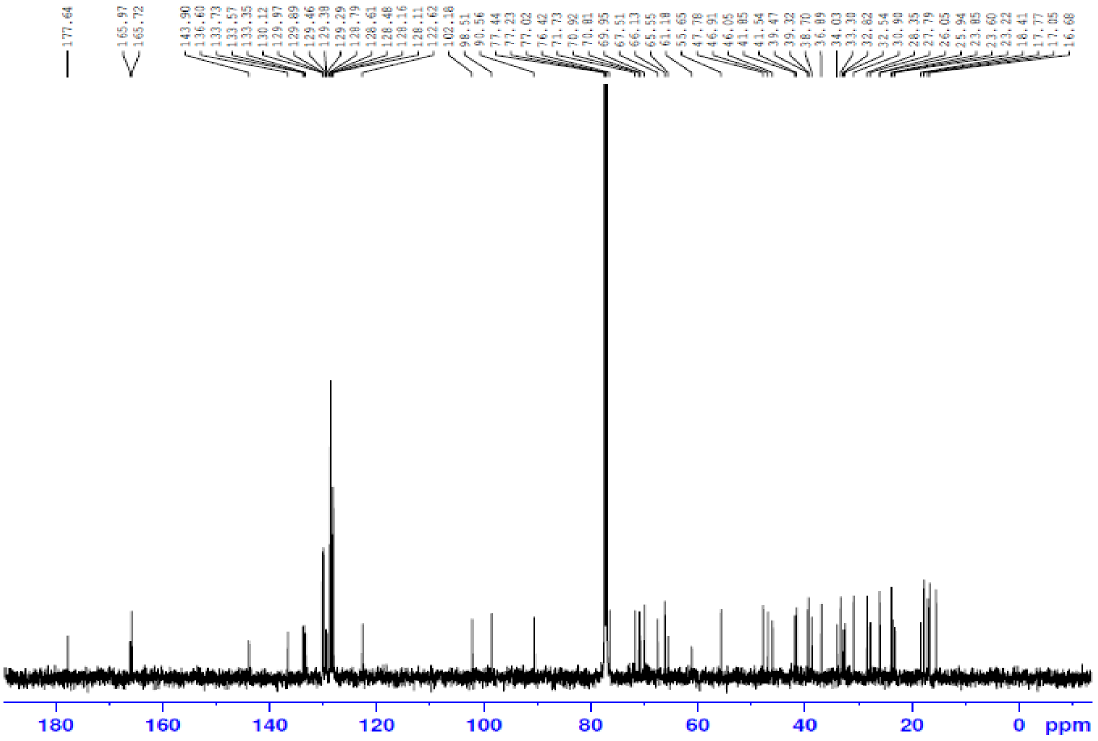

Figure S10. COSY of compound **7**.

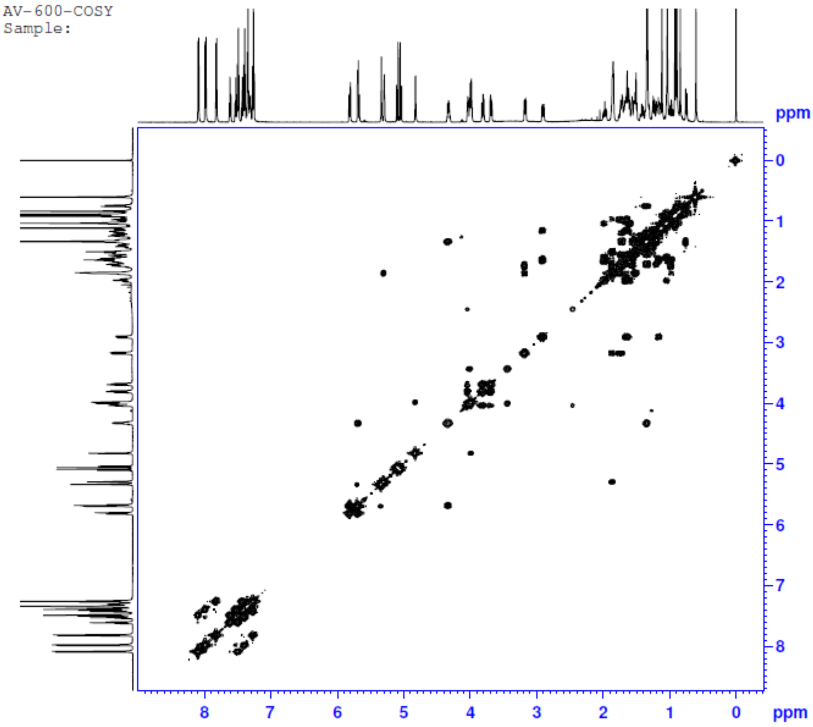

Figure S11. HSQC of compound **7**.

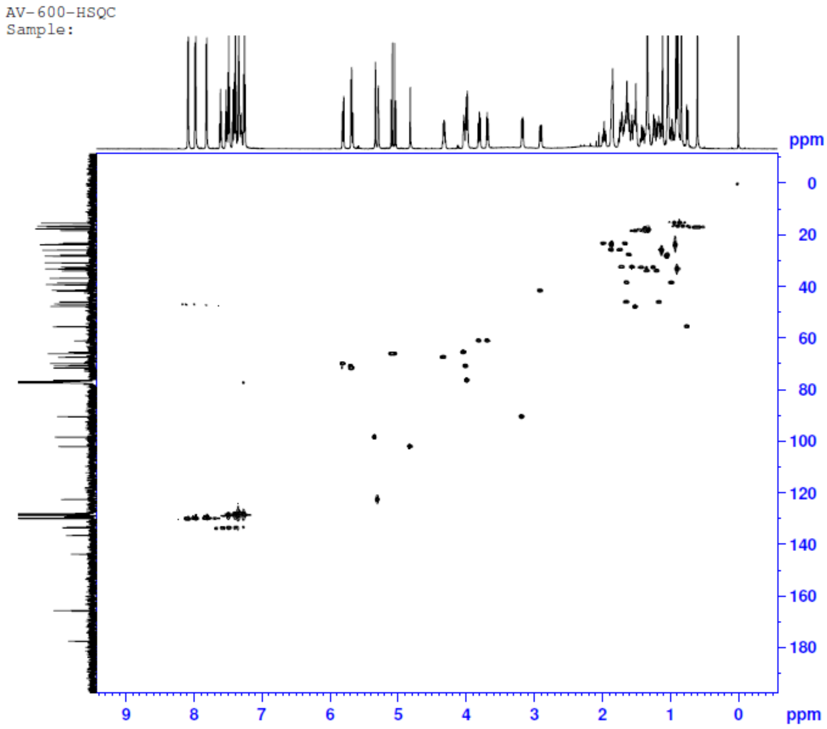

Figure S12. HMBC of compound **7**.

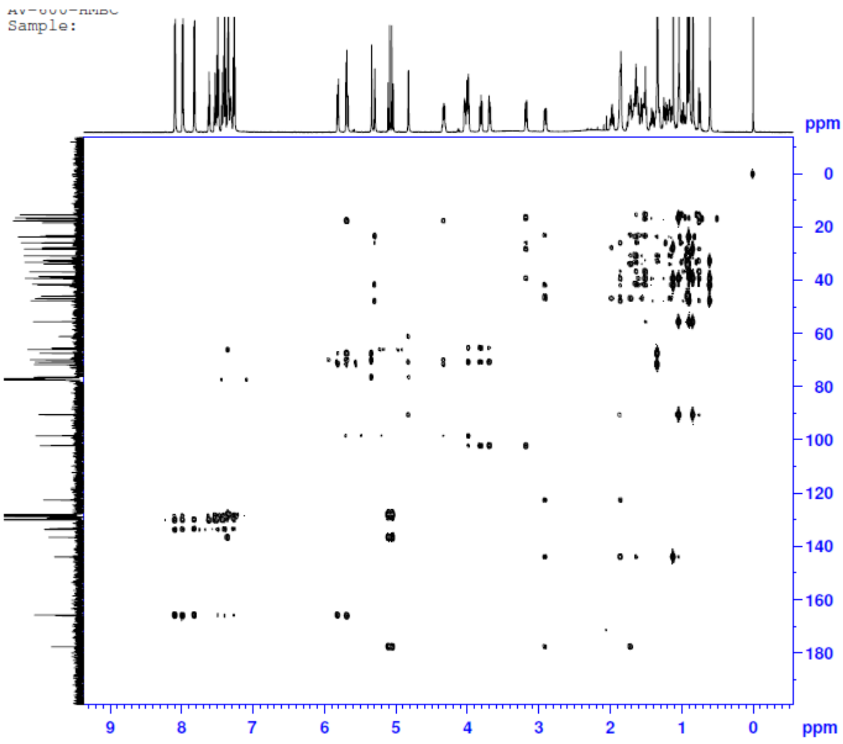

Figure S13. Structure of compound **8**.

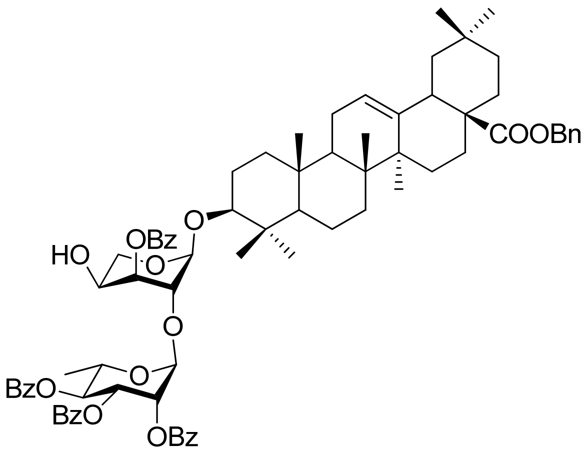

Figure S14. ^{1}H-NMR of compound **8**.

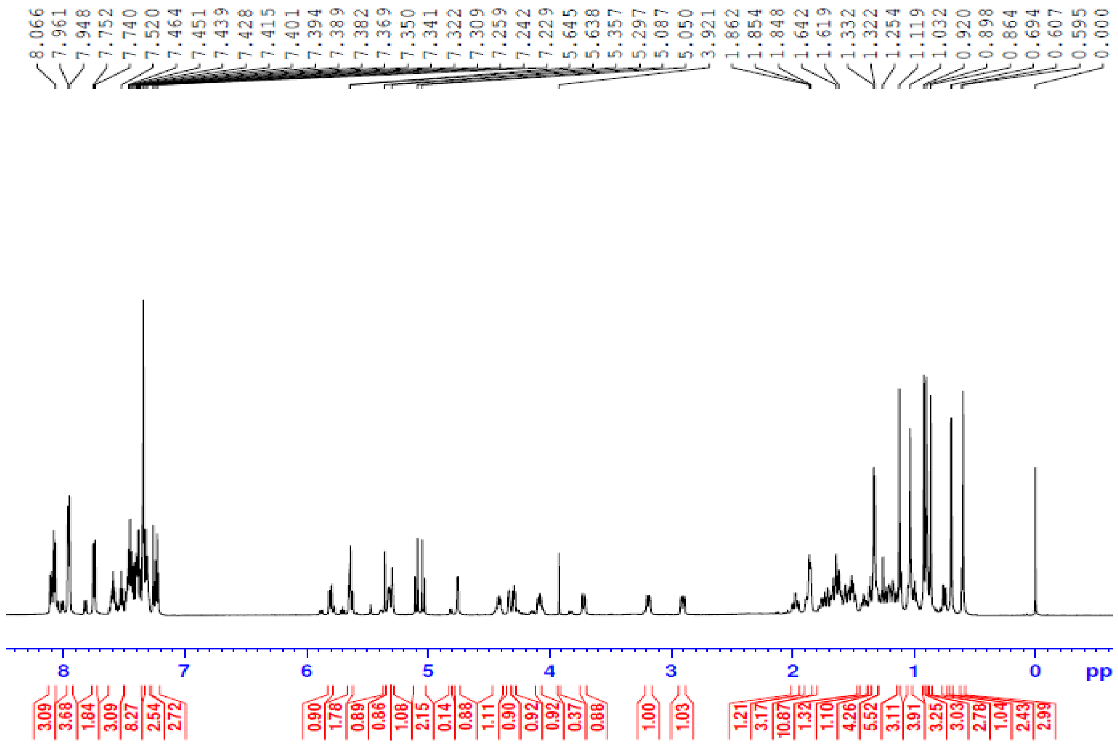

Figure S15. ^{13}C-NMR of compound **8**.

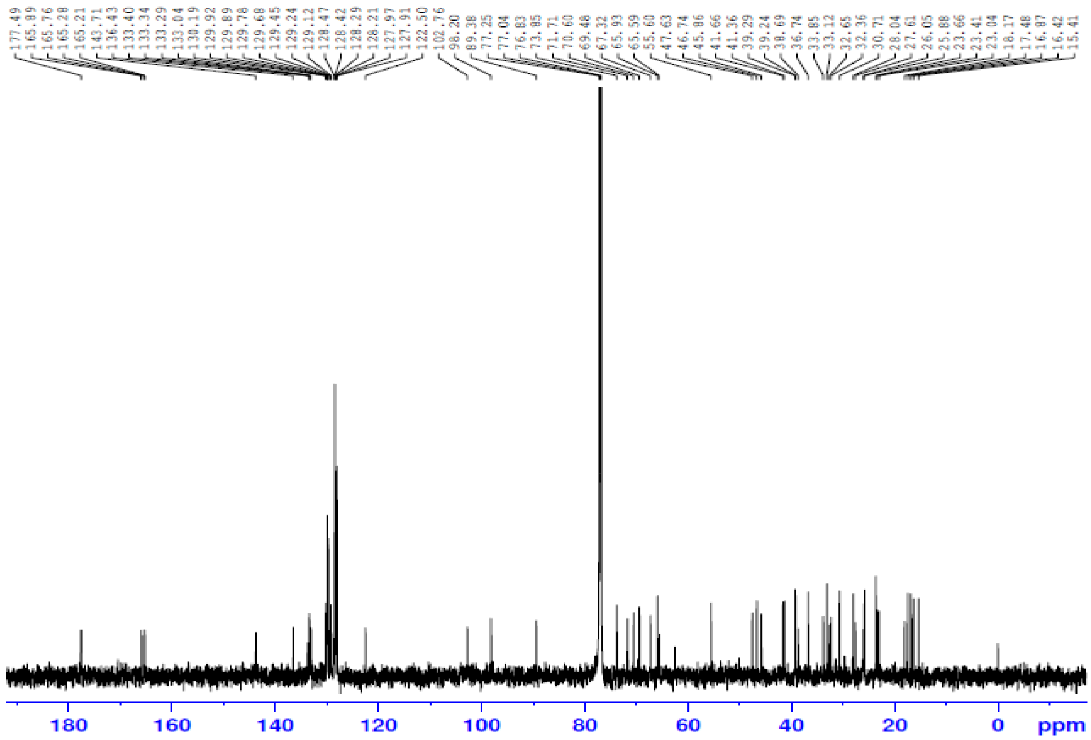

Figure S16. COSY of compound **8**.

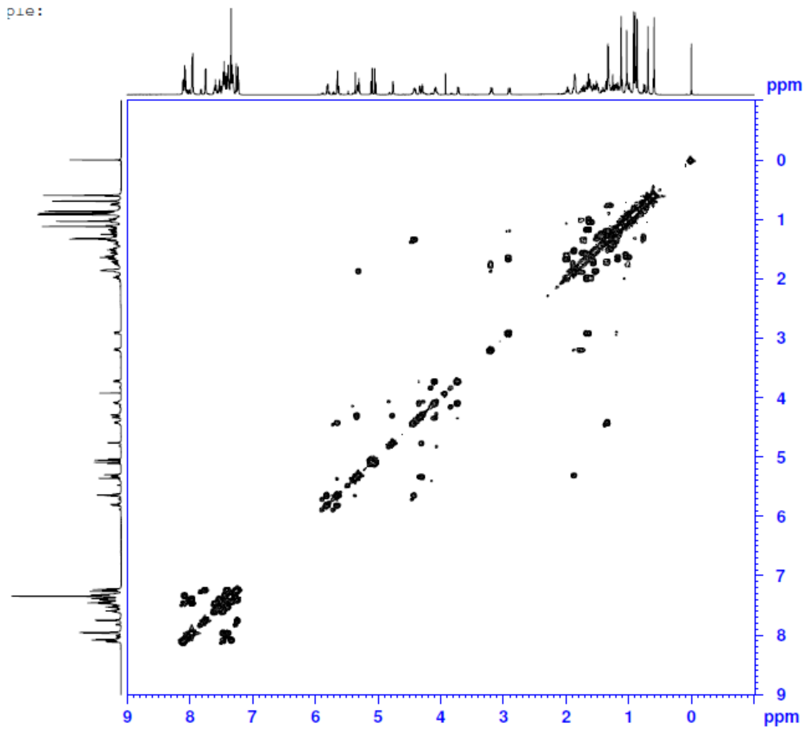

Figure S17. HSQC of compound **8**.

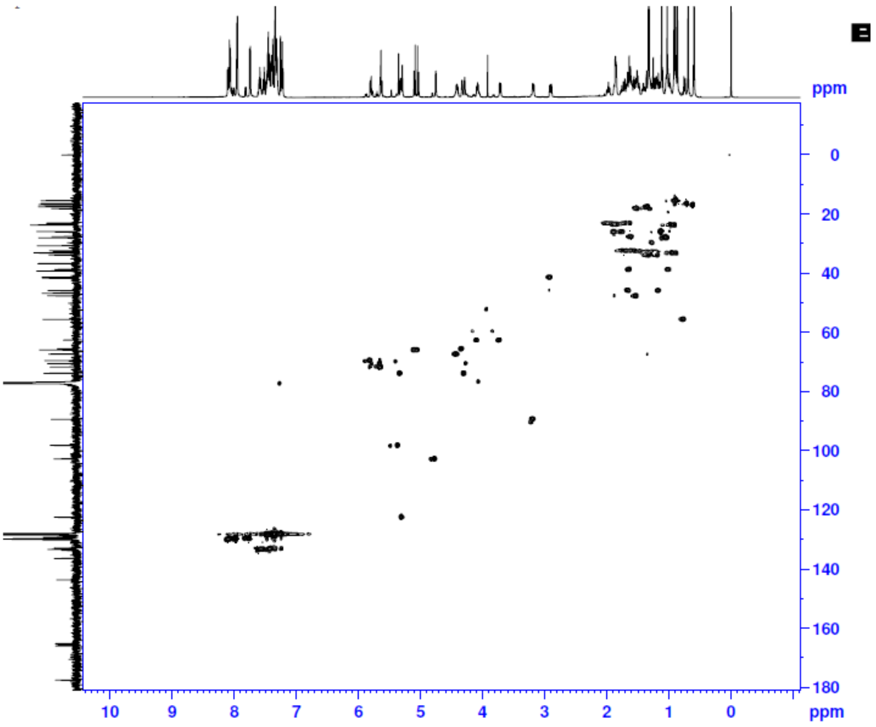

Figure S18. HMBC of compound **8**.

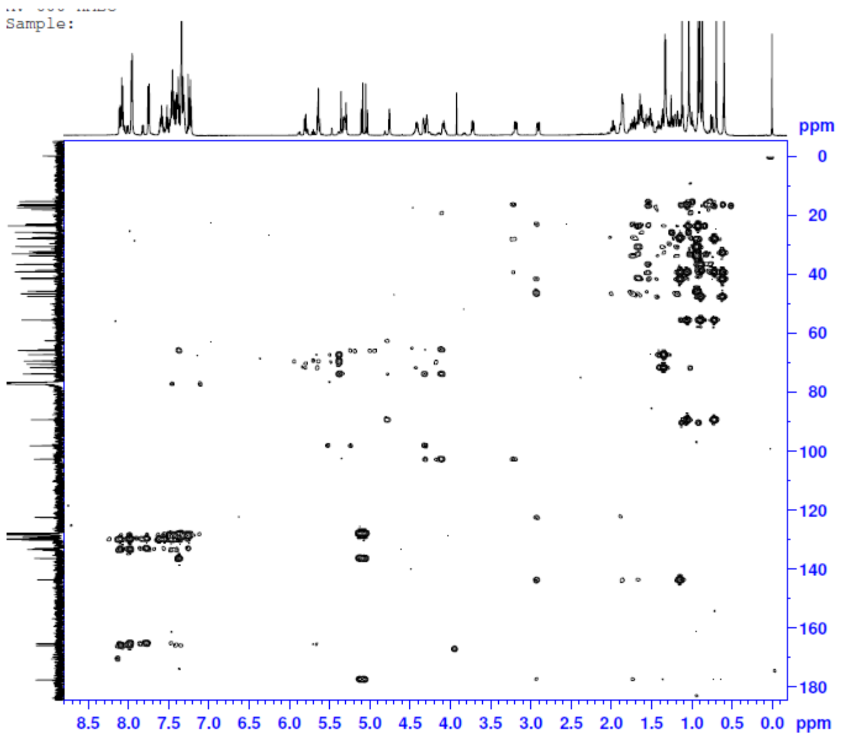

Figure S19. Structure of compound **9**.

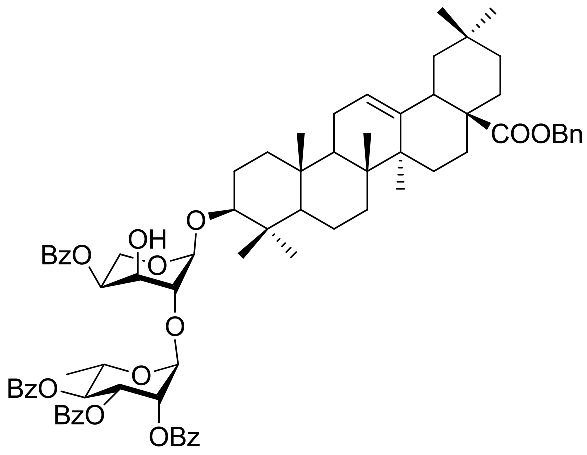

Figure S20. ^{1}H-NMR of compound **9**.

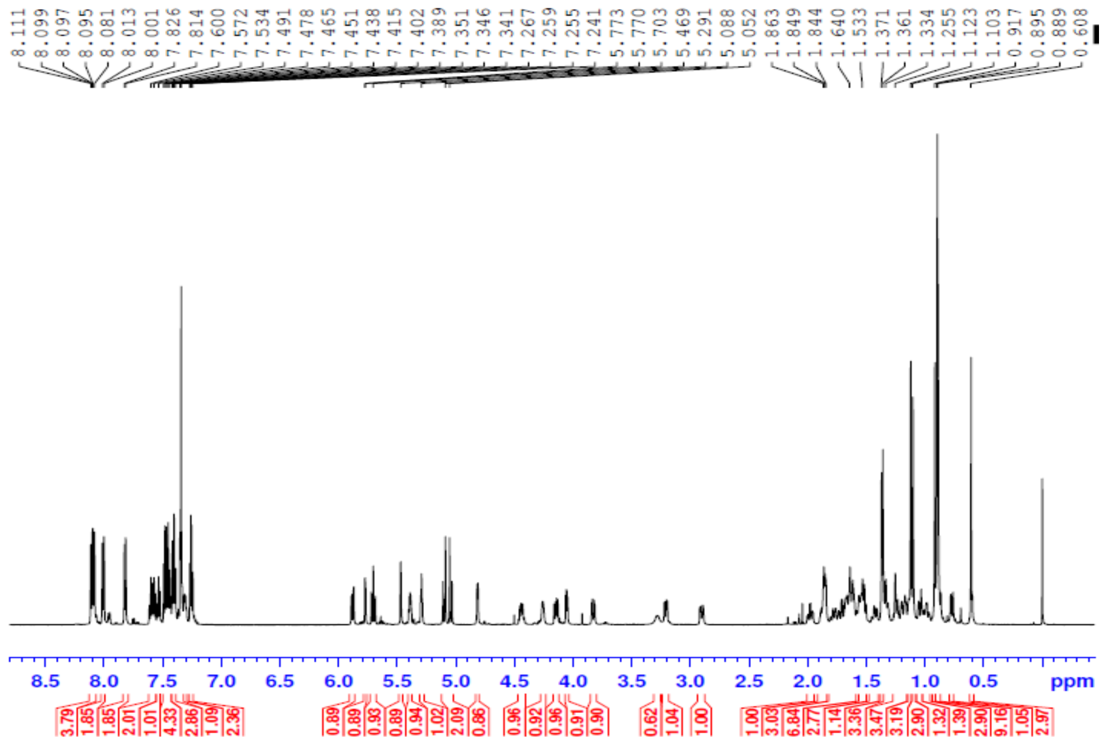

Figure S21. ^{13}C-NMR of compound **9**.

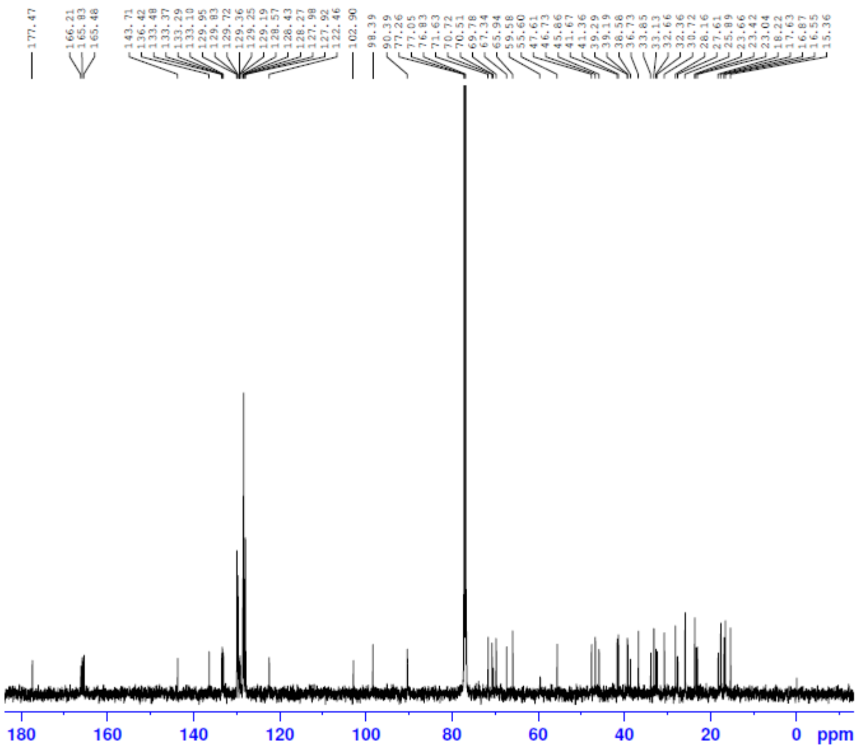

Figure S22. COSY of compound **9**.

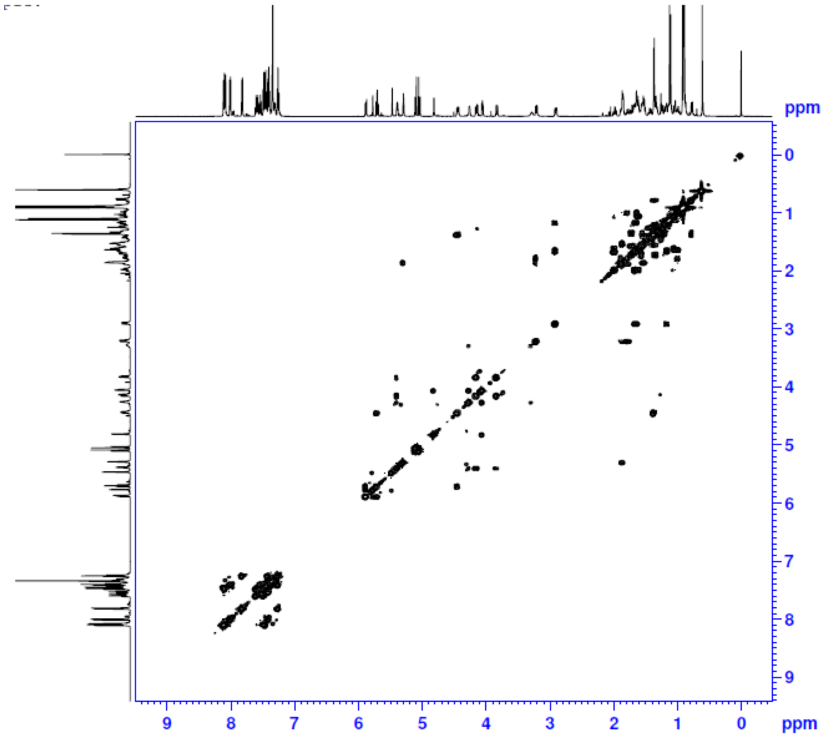

Figure S23. HSQC of compound **9**.

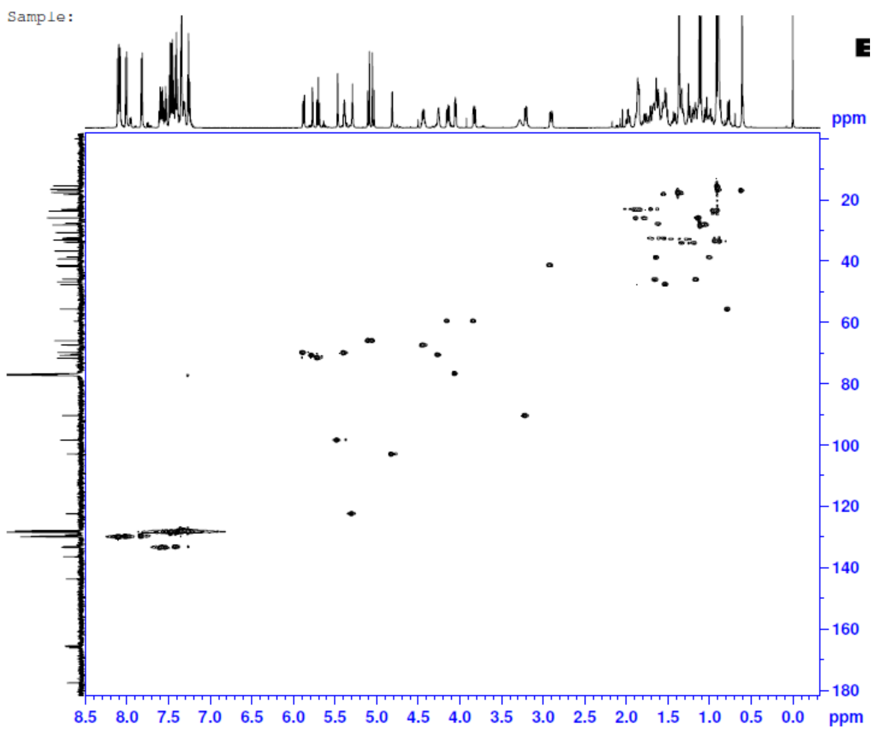

Figure S24. HMBC of compound **9**.

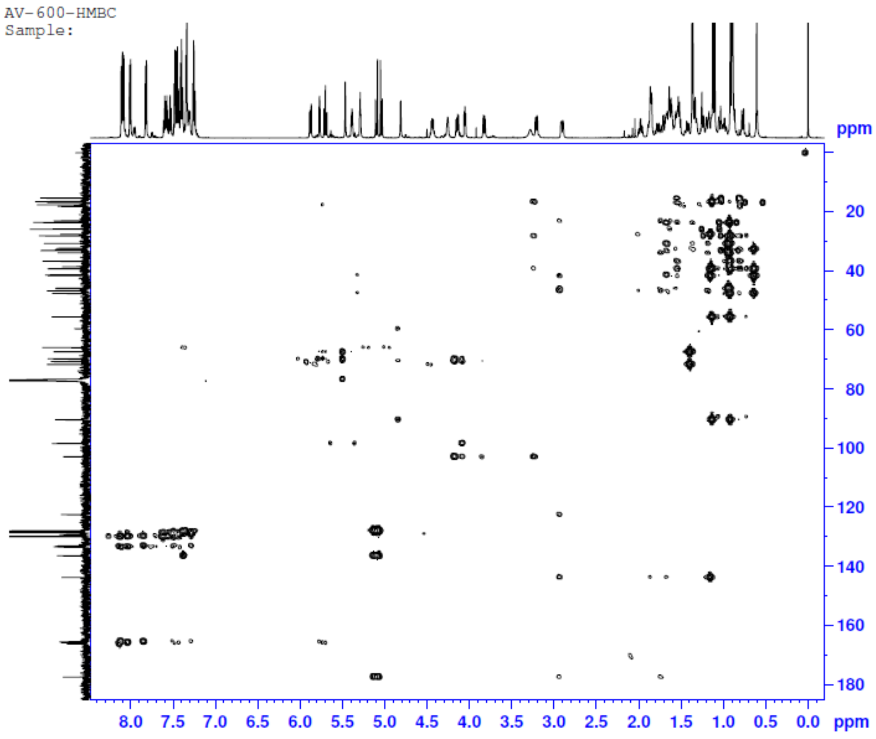

Figure S25. Structure of compound **1**.

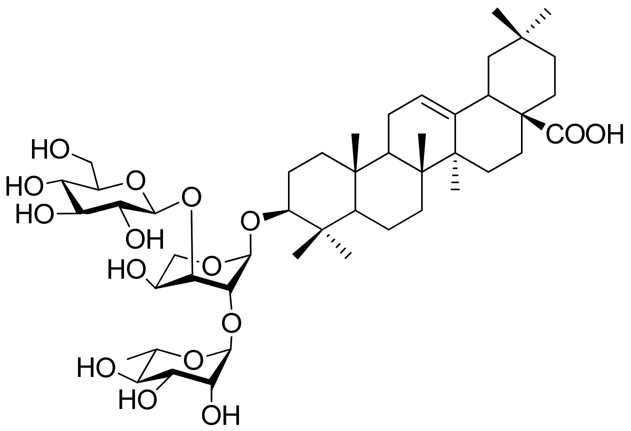

Figure S26. MS of compound **1**.

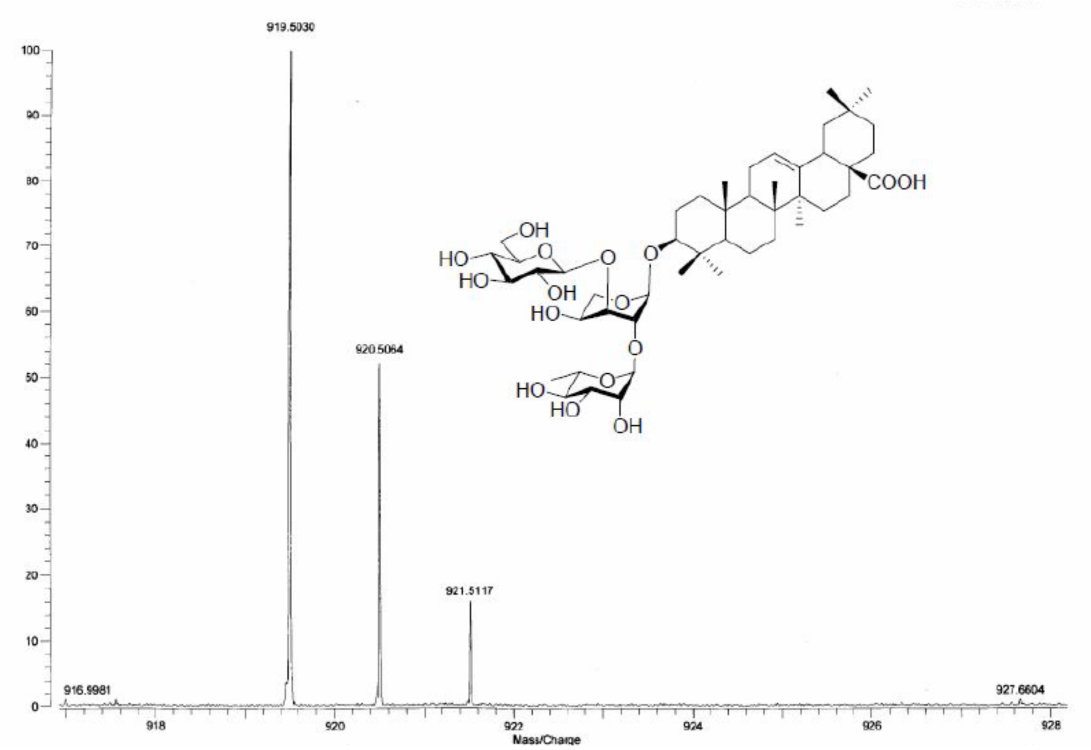

Figure S27. ^{1}H-NMR of compound **1**.

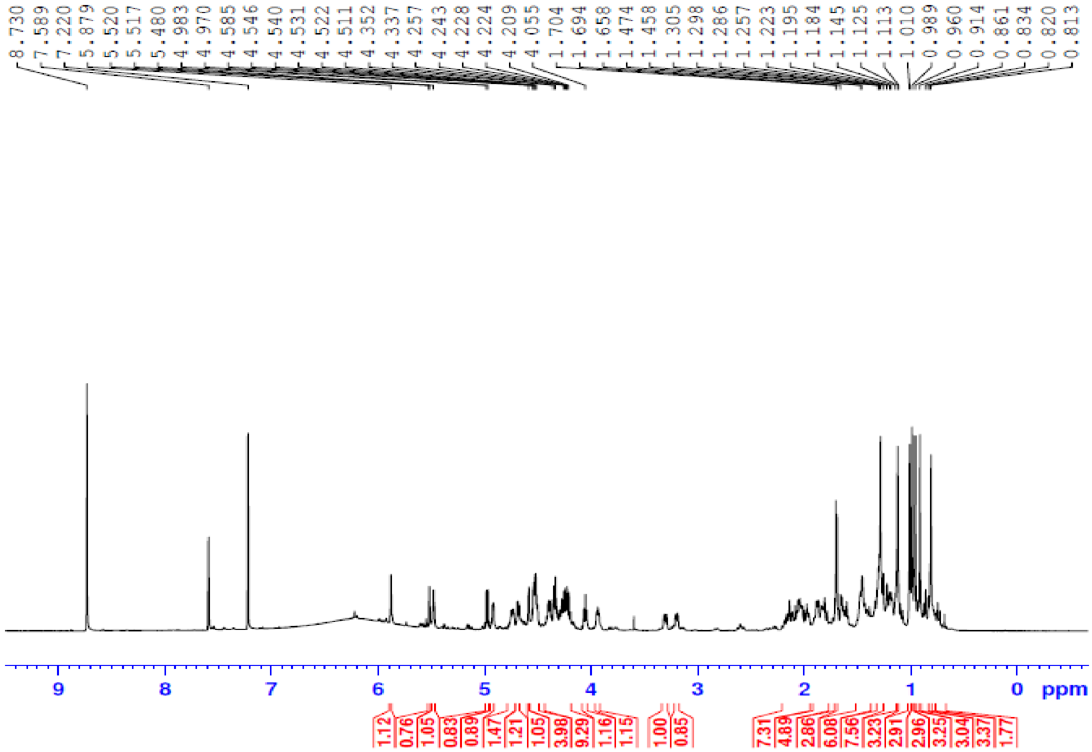

Figure S28. ^{13}C-NMR of compound **1**.

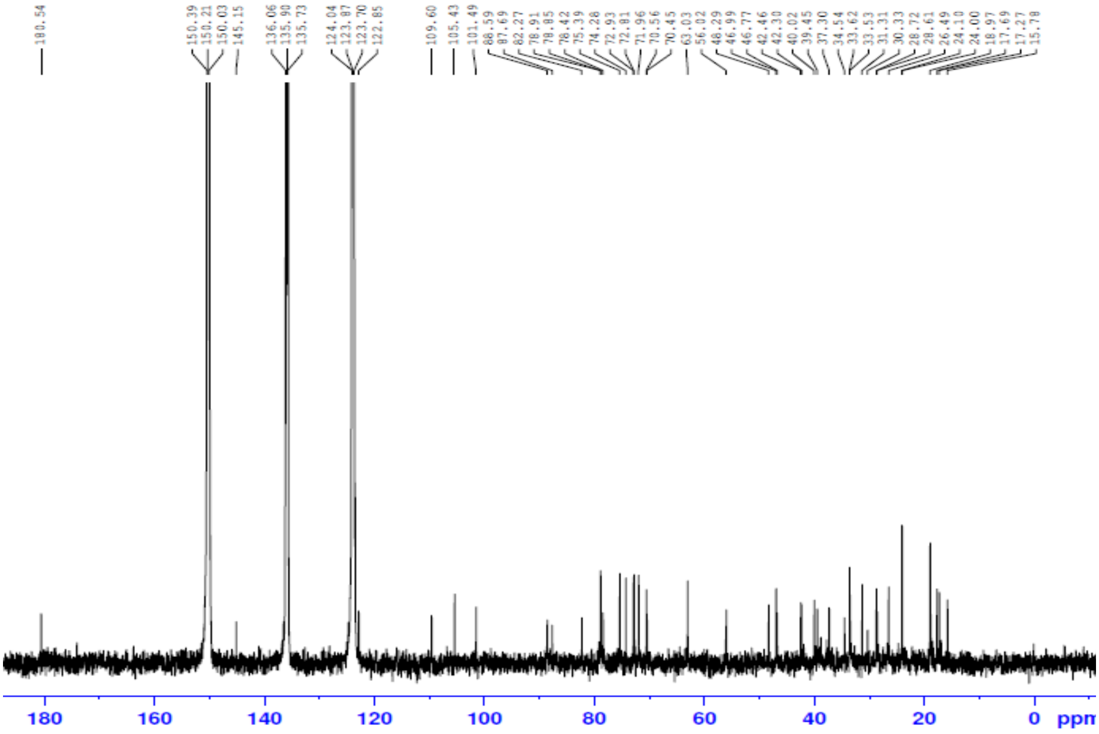

Figure S29. COSY of compound **1**.

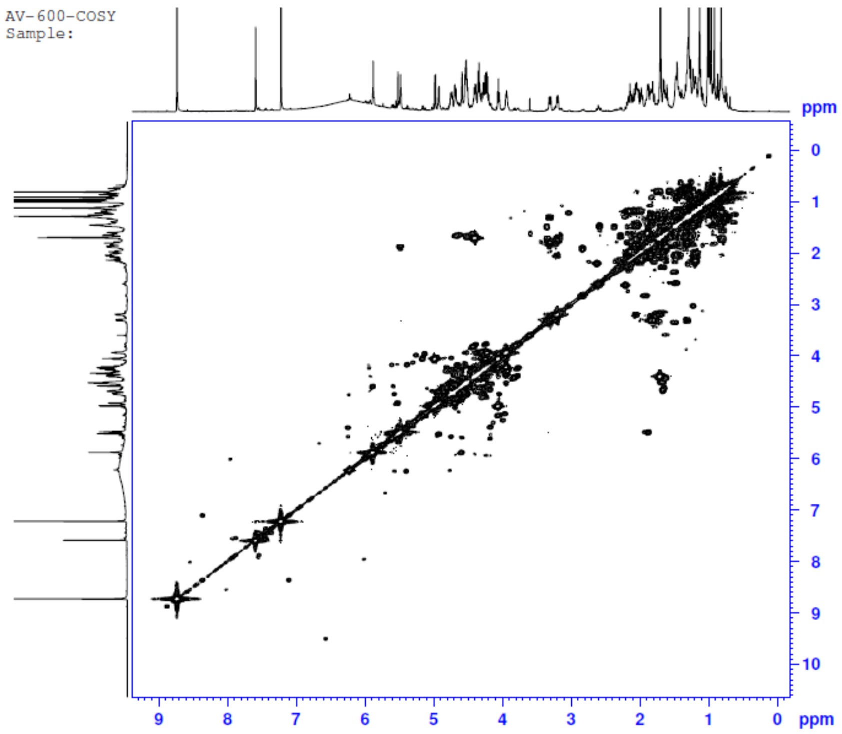

Figure S30. HSQC of compound **1**.

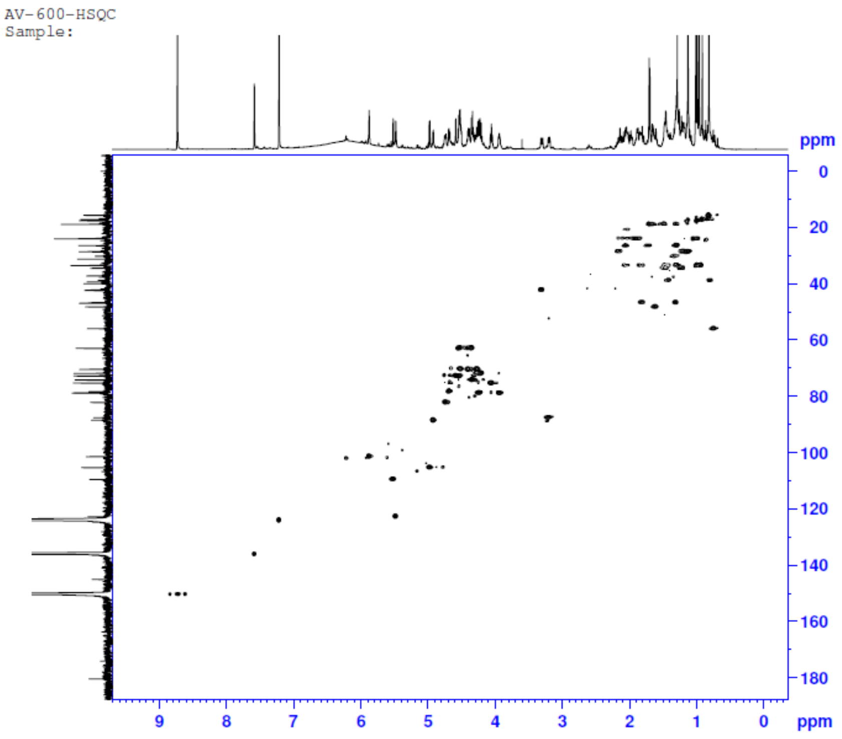

Figure S31. HMBC of compound **1**.

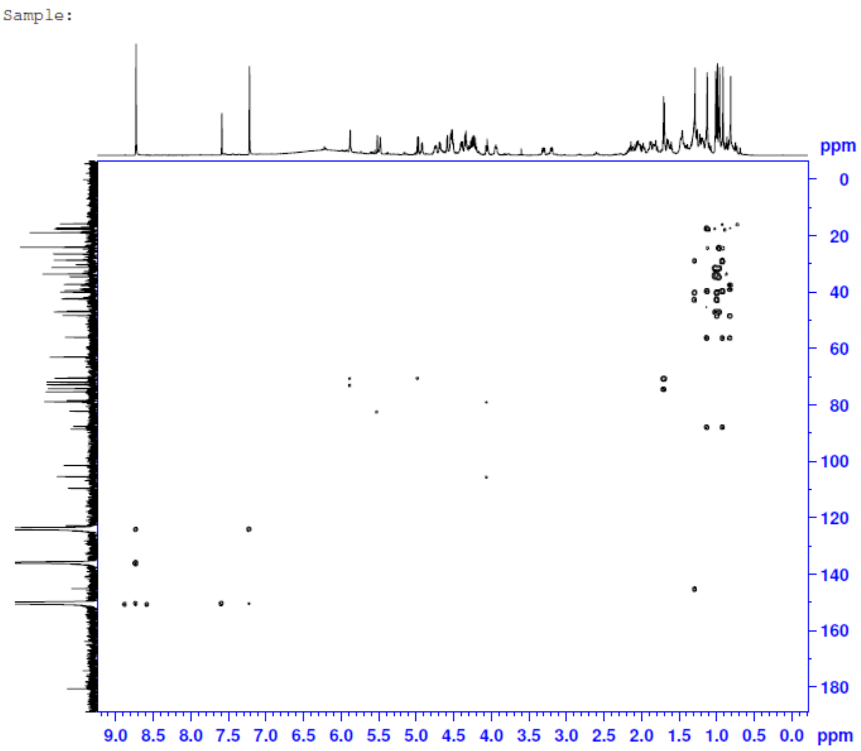

Figure S32. NOESY of compound **1**.

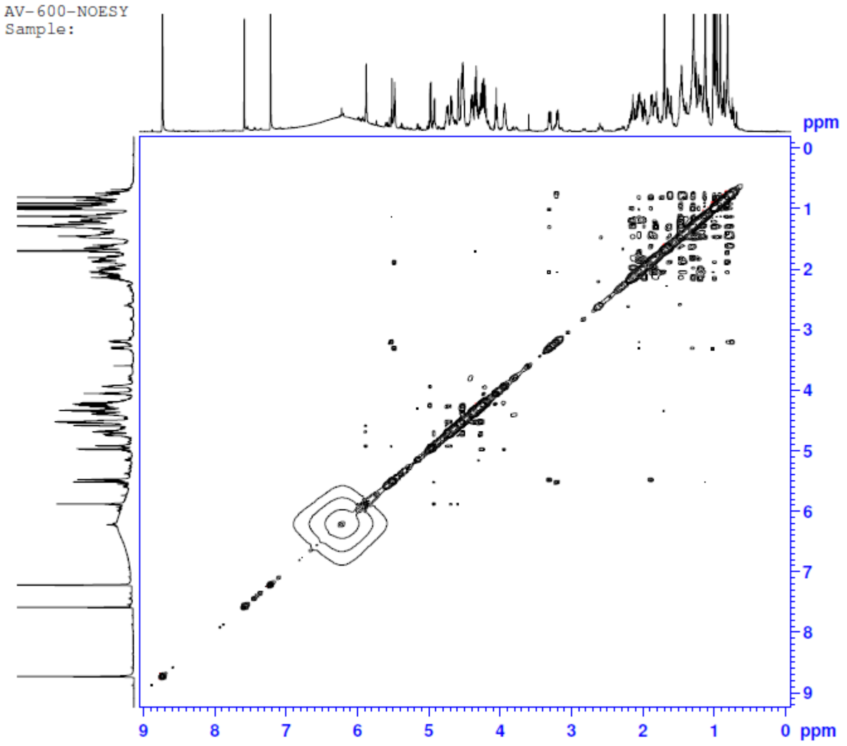

Figure S33. Structure of compound **11**.

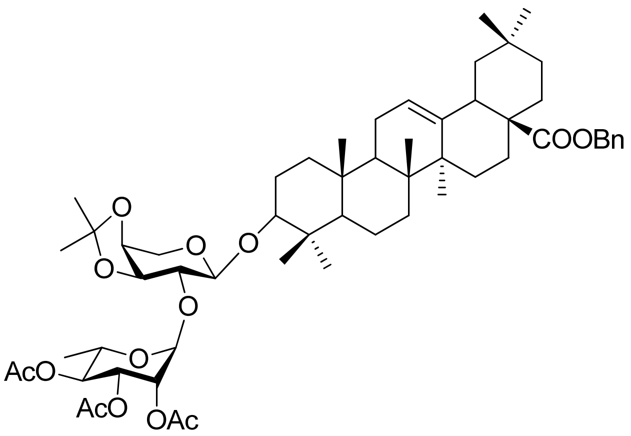

Figure S34. ^{1}H-NMR of compound **11**.

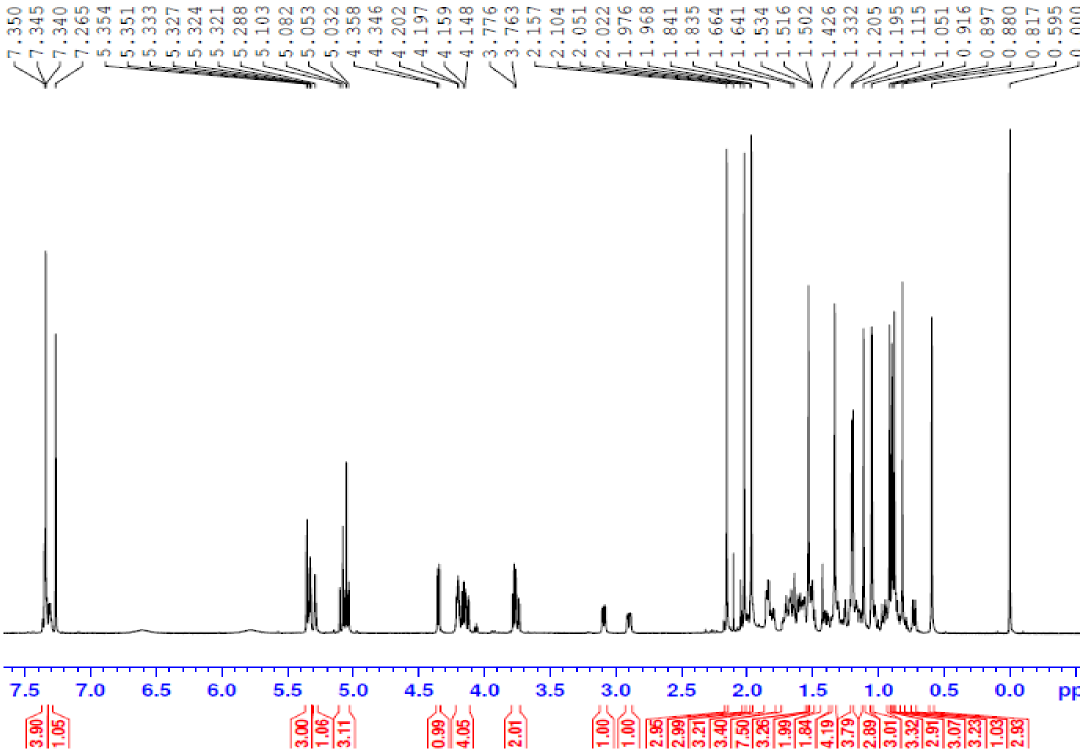

Figure S35. ^{13}C-NMR of compound **11**.

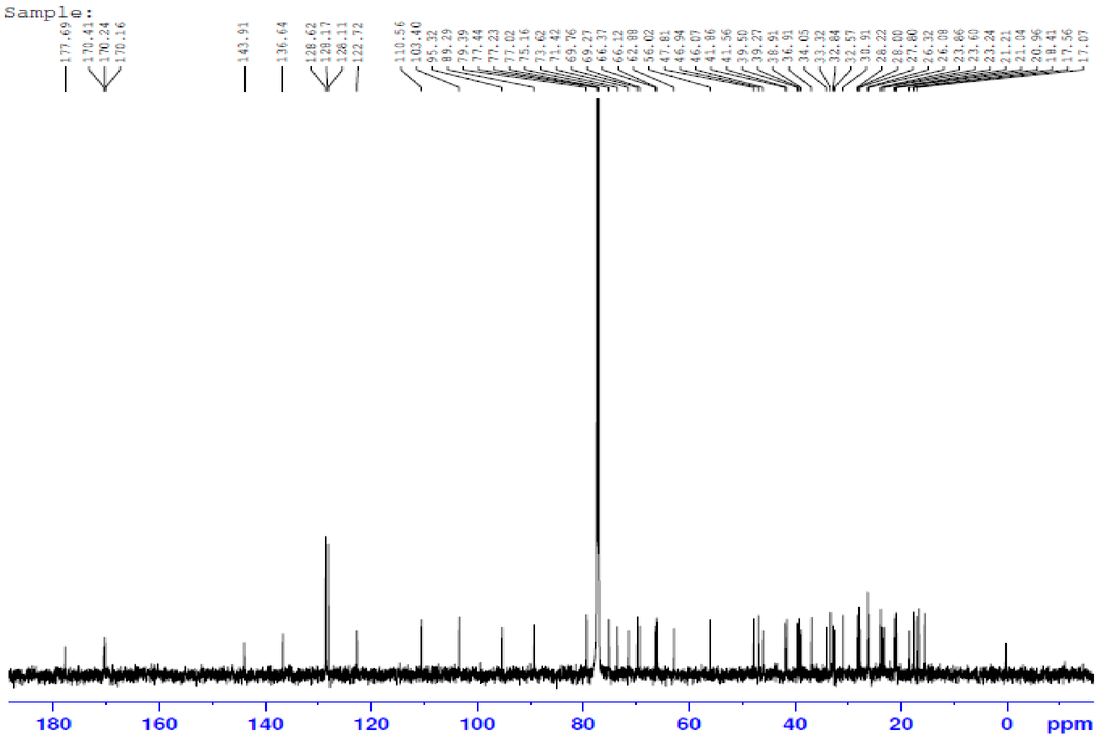

Figure S36. COSY of compound **11**.

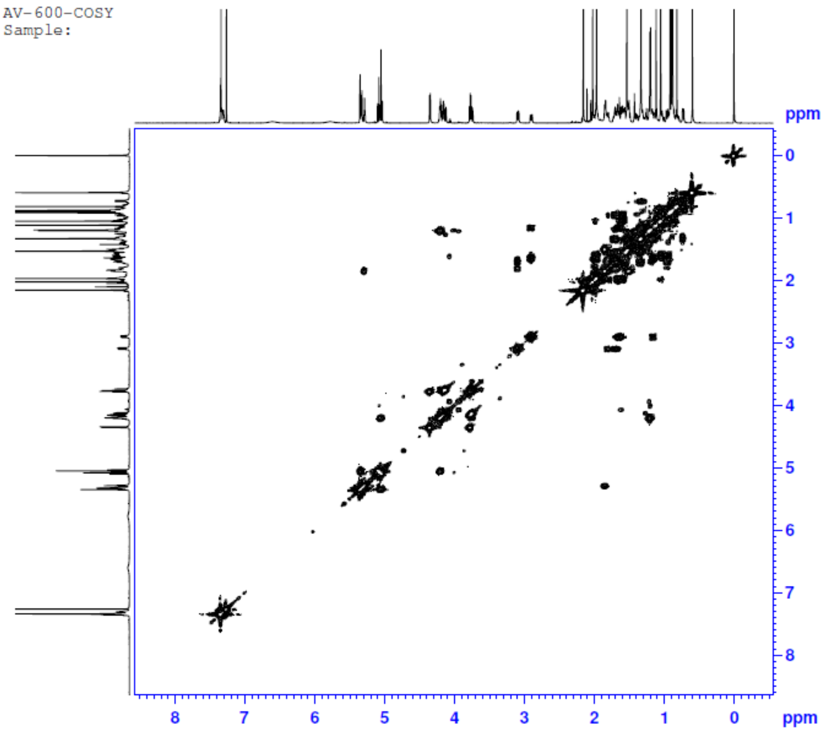

Figure S37. HSQC of compound **11**.

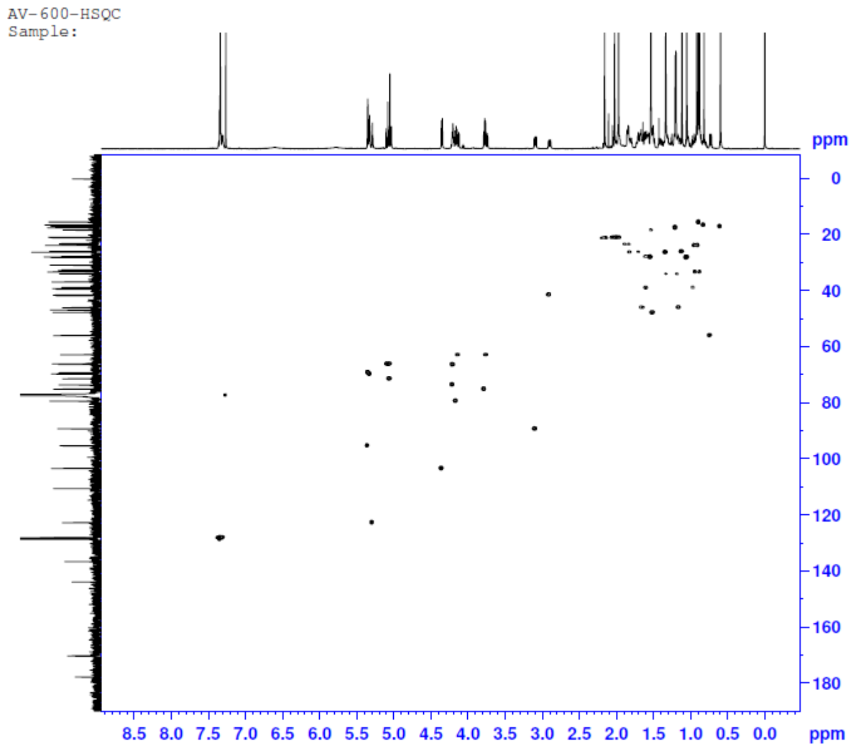

Figure S38. HMBC of compound **11**.

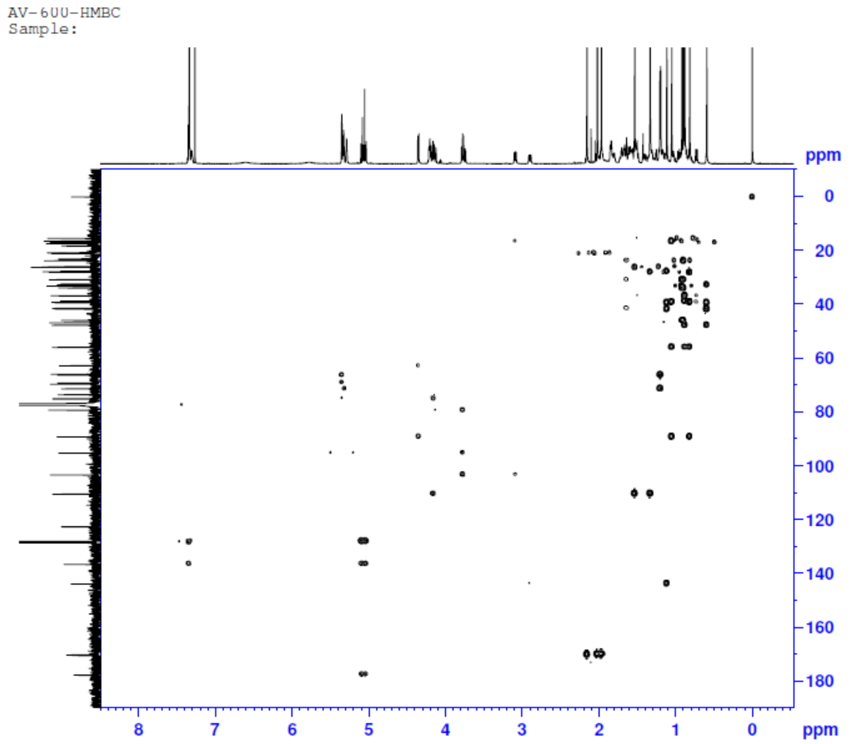

Figure S39. Structure of compound **12**.

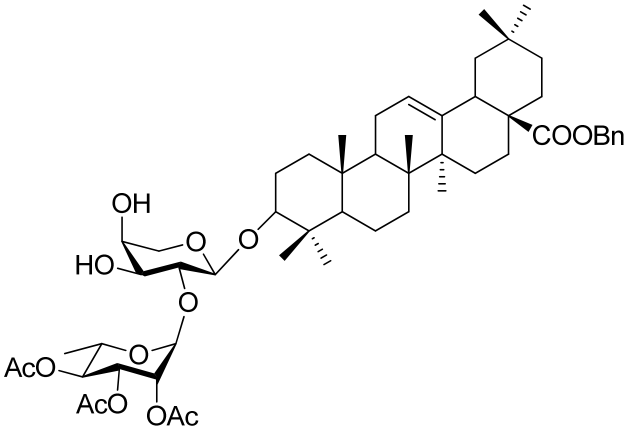

Figure S40. ^{1}H-NMR of compound **12**.

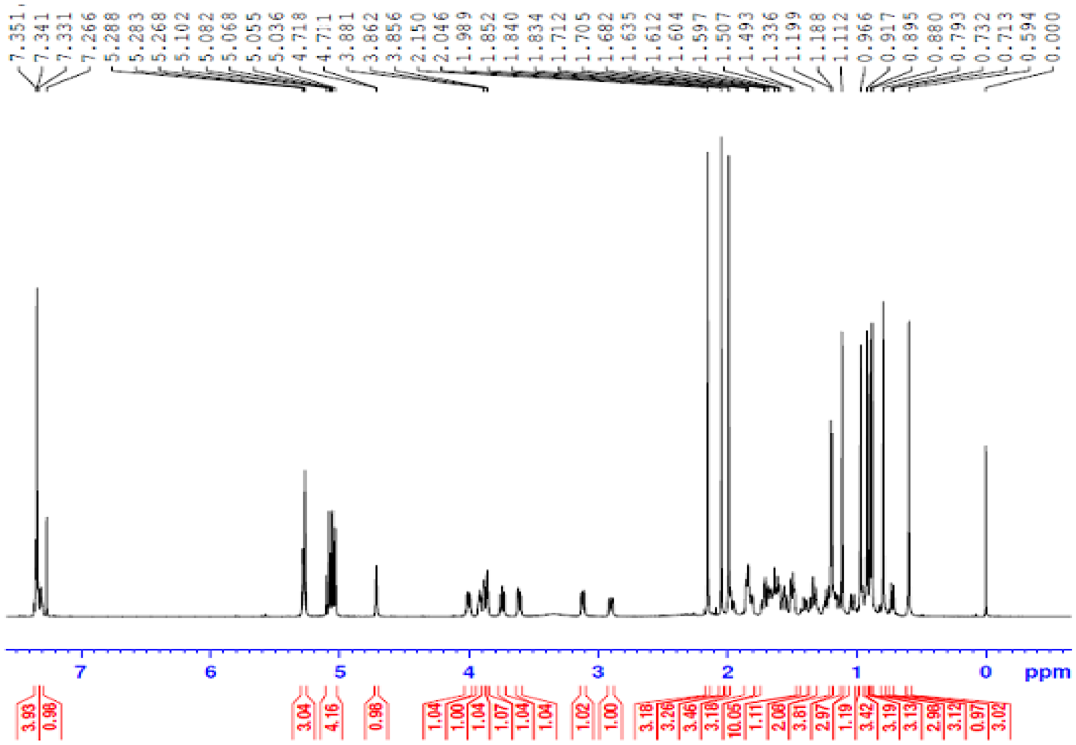

Figure S41. ^{13}C-NMR of compound **12**.

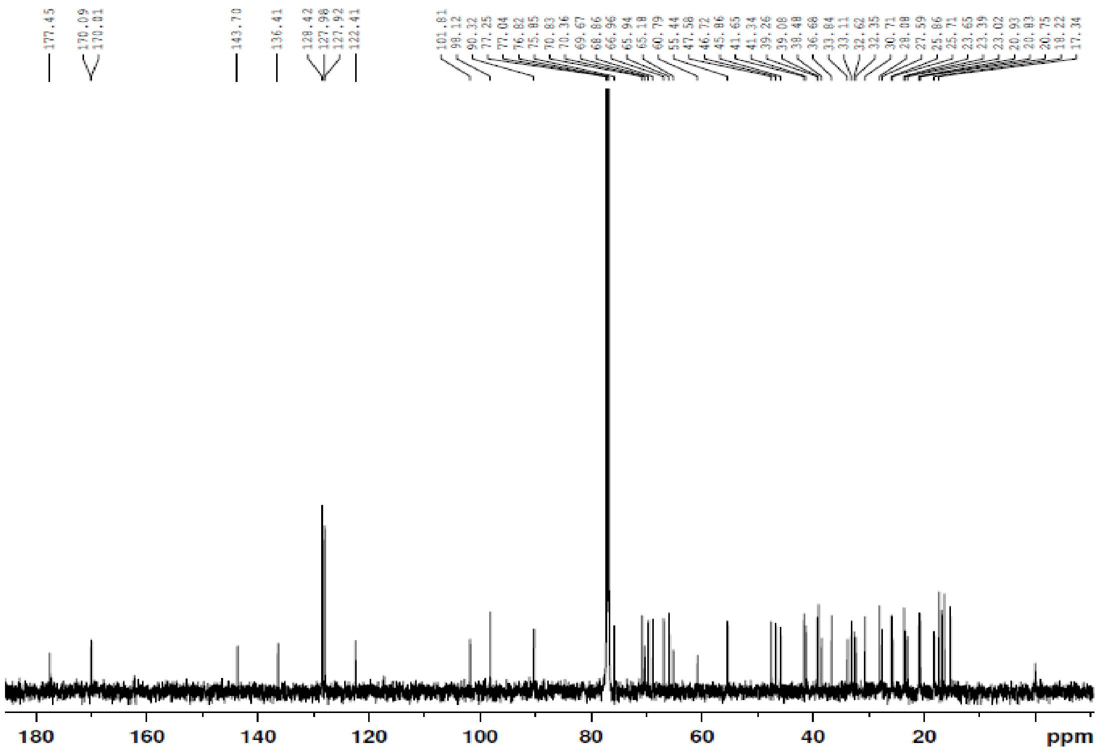

Figure S42. COSY of compound **12**.

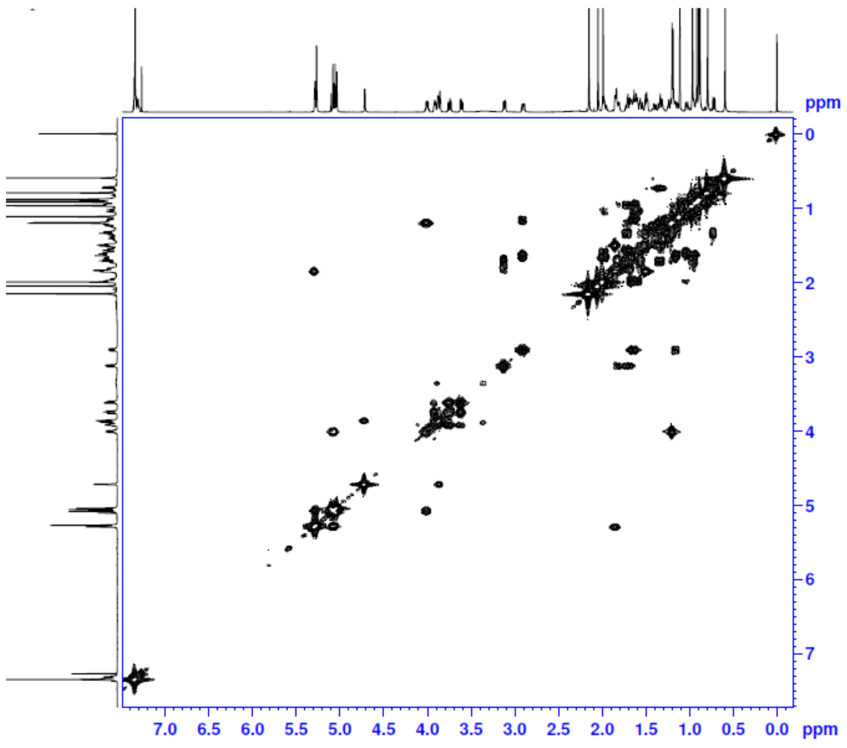

Figure S43. HSQC of compound **12**.

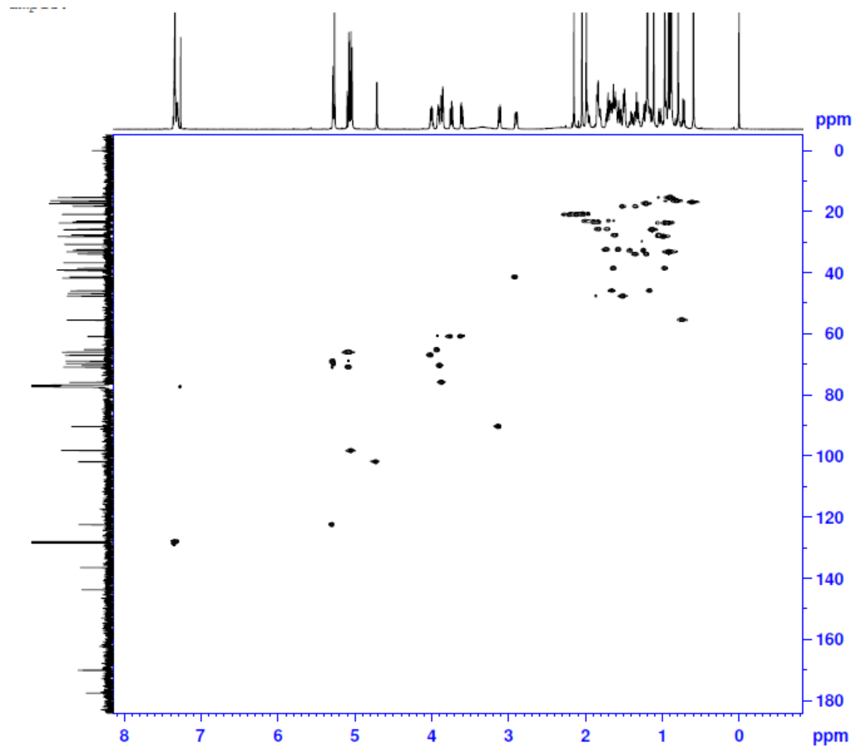

Figure S44. HMBC of compound **12**.

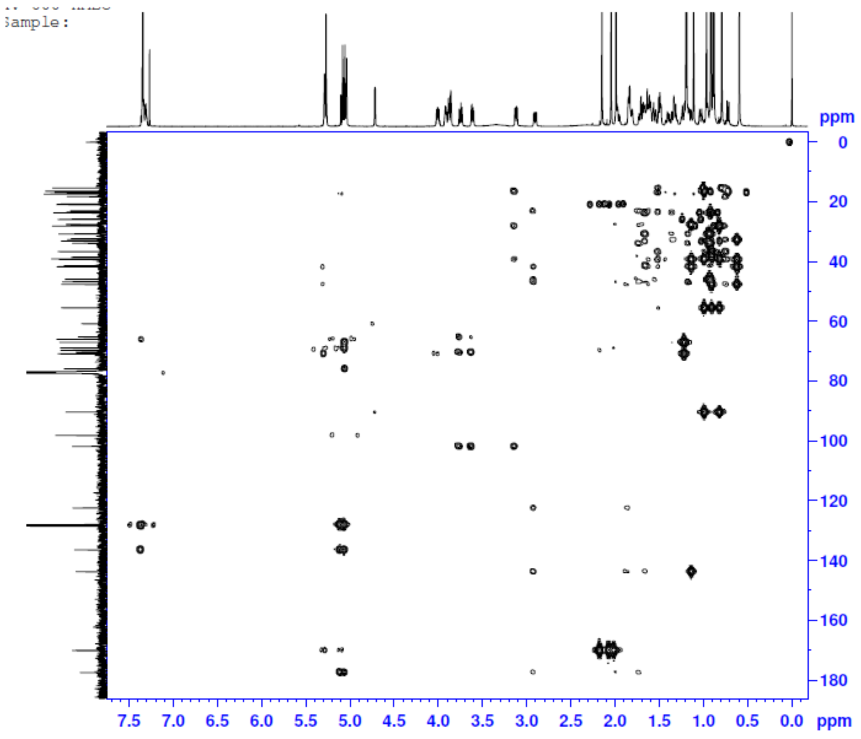

Figure S45. Structure of compound **13**.

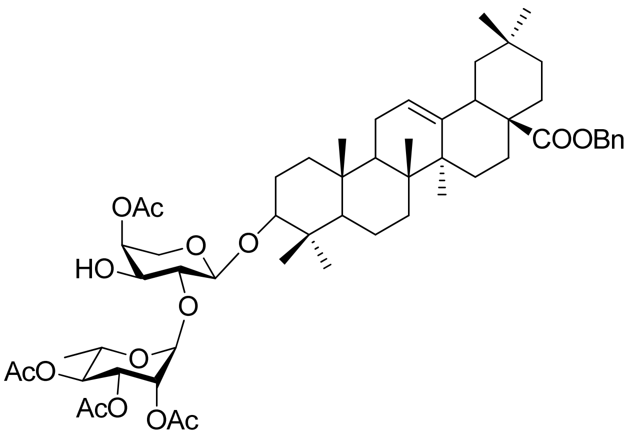

Figure S46. ^{1}H-NMR of compound **13**.

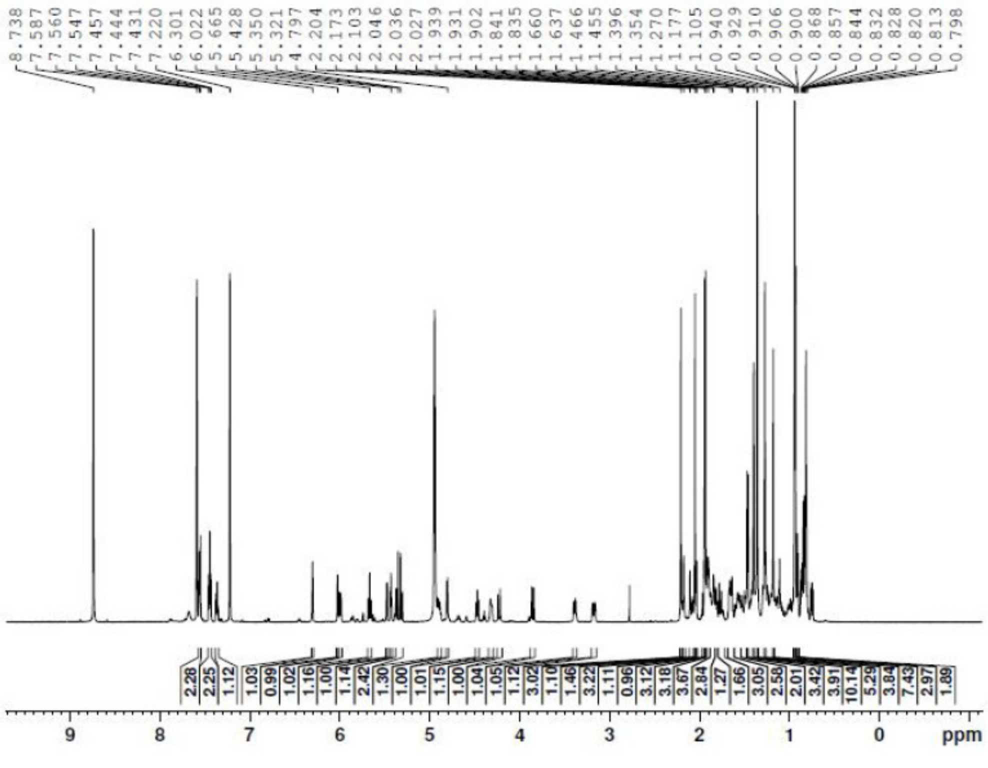

Figure S47. ^{13}C-NMR of compound**13**.

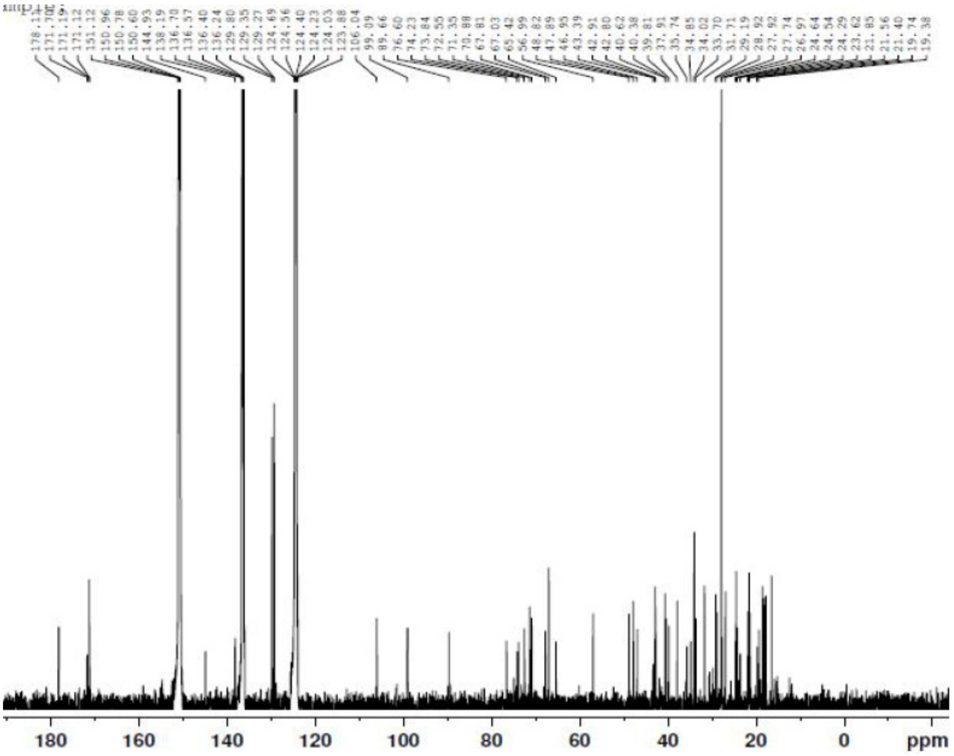

Figure S48. COSY of compound **13**.

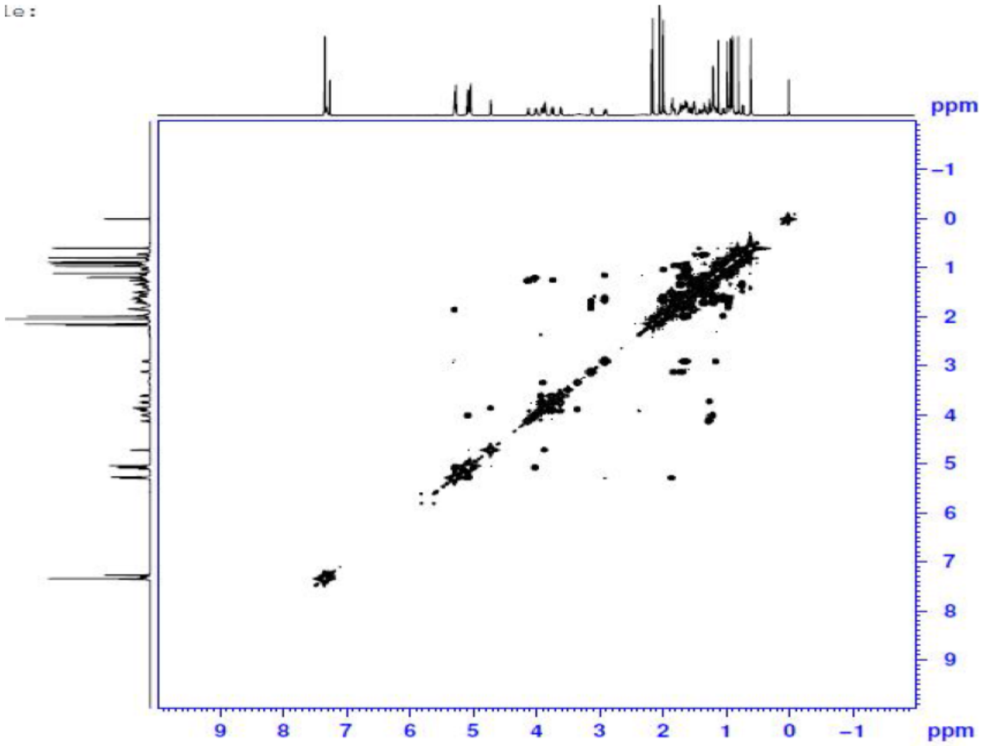

Figure S49. HSQC of compound **13**.

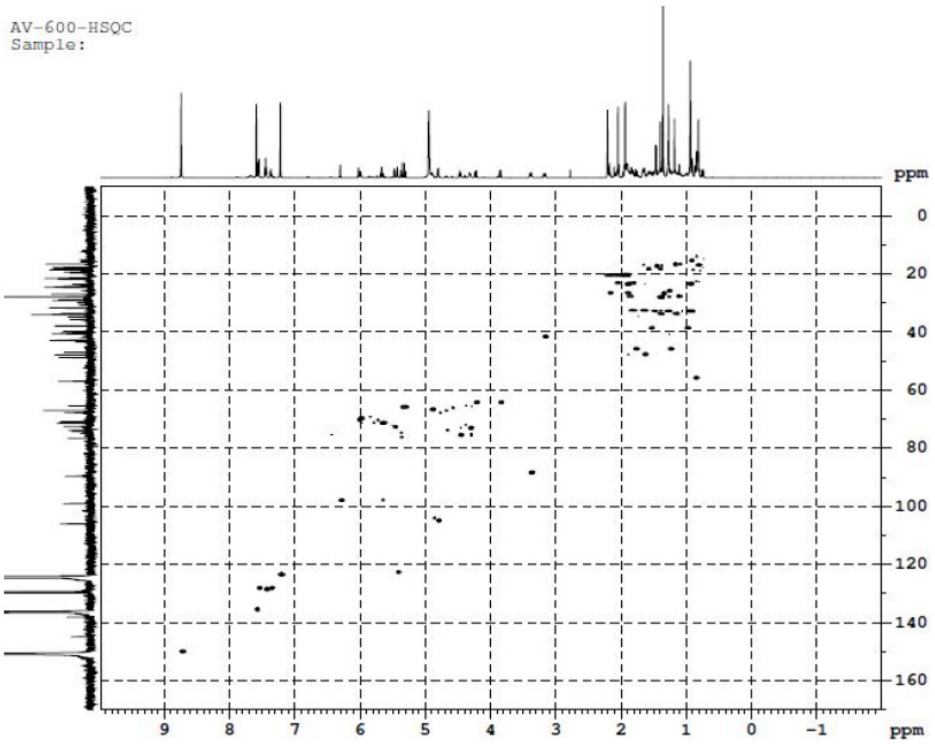

Figure S50. Structure of Patrinia-glycoside B-II.

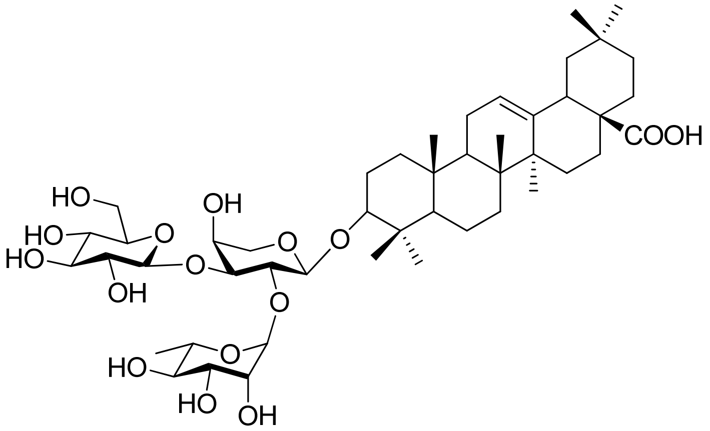

Figure S51. MS of Patrinia-glycoside B-II.

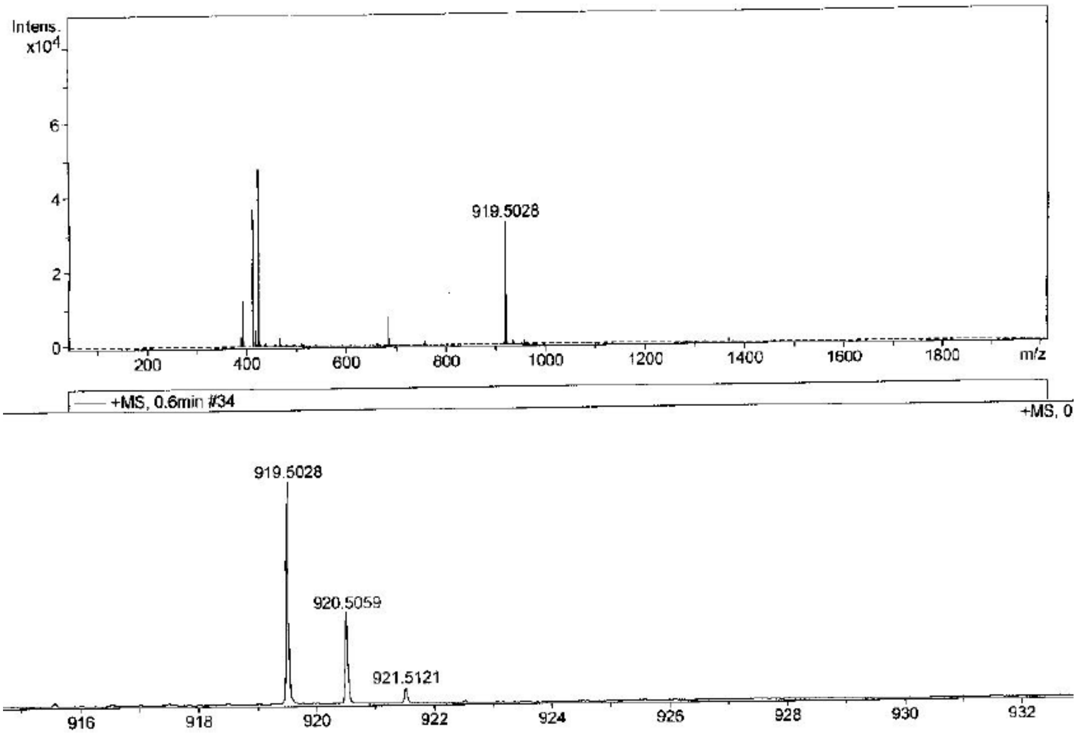

Figure S52. ^{1}H-NMR of Patrinia-glycoside B-II.

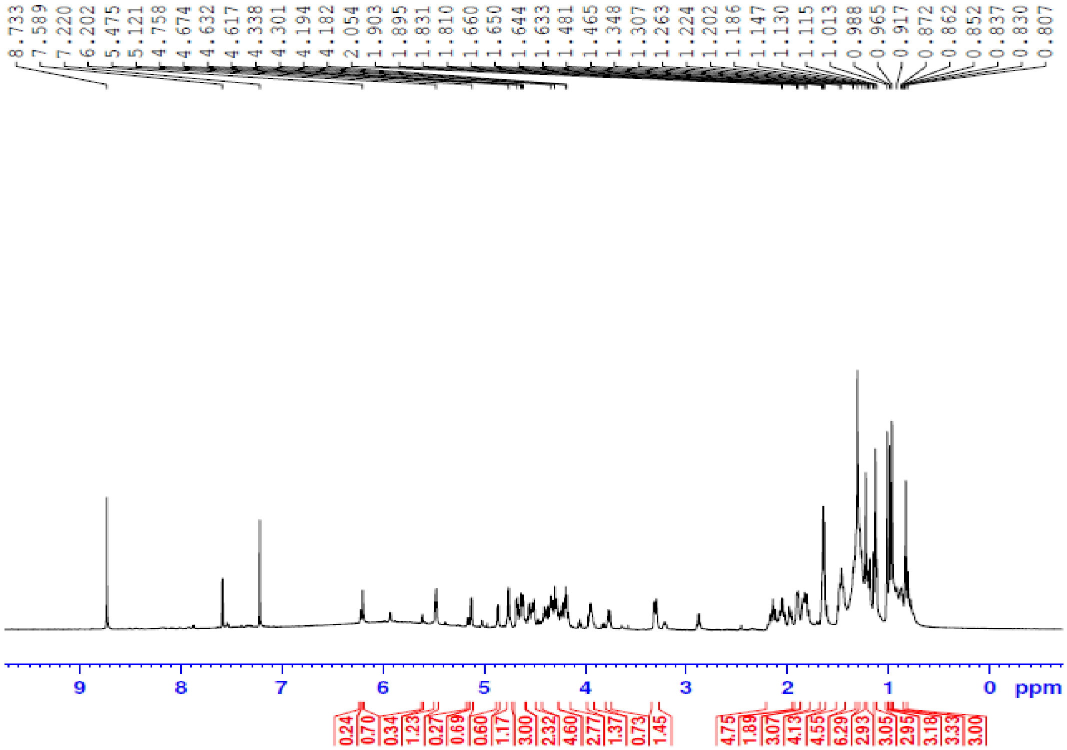

Figure S53. ^{13}C-NMR of Patrinia-glycoside B-II.

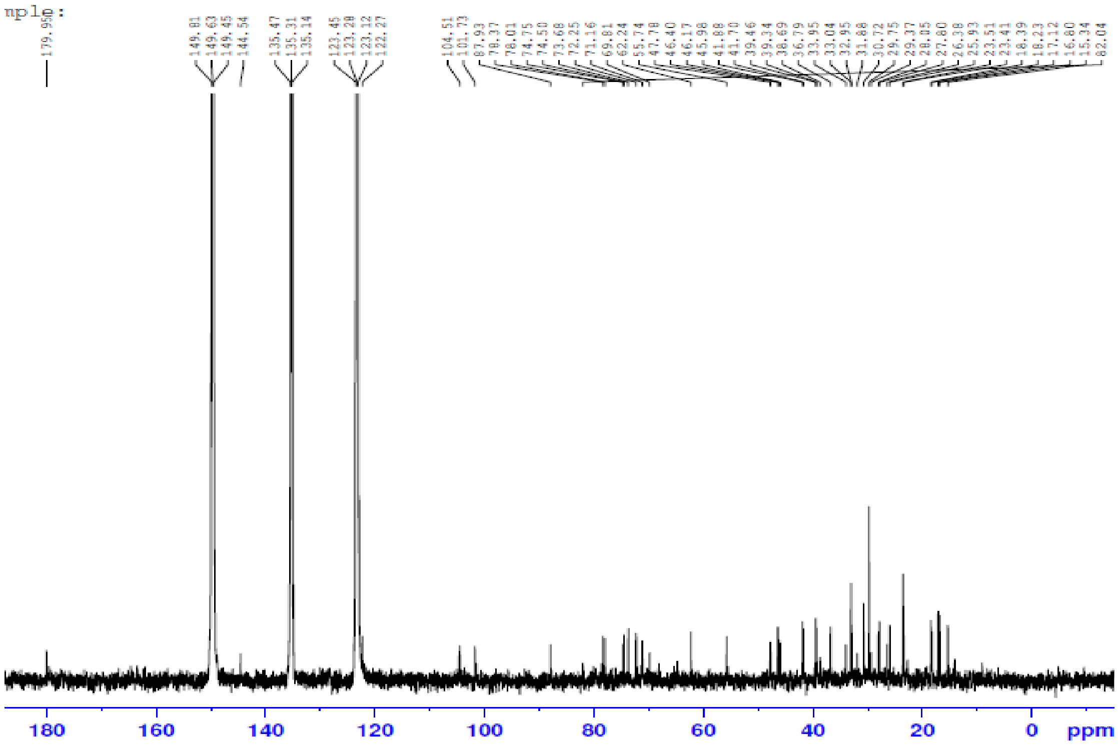

Figure S54. COSY of Patrinia-glycoside B-II.

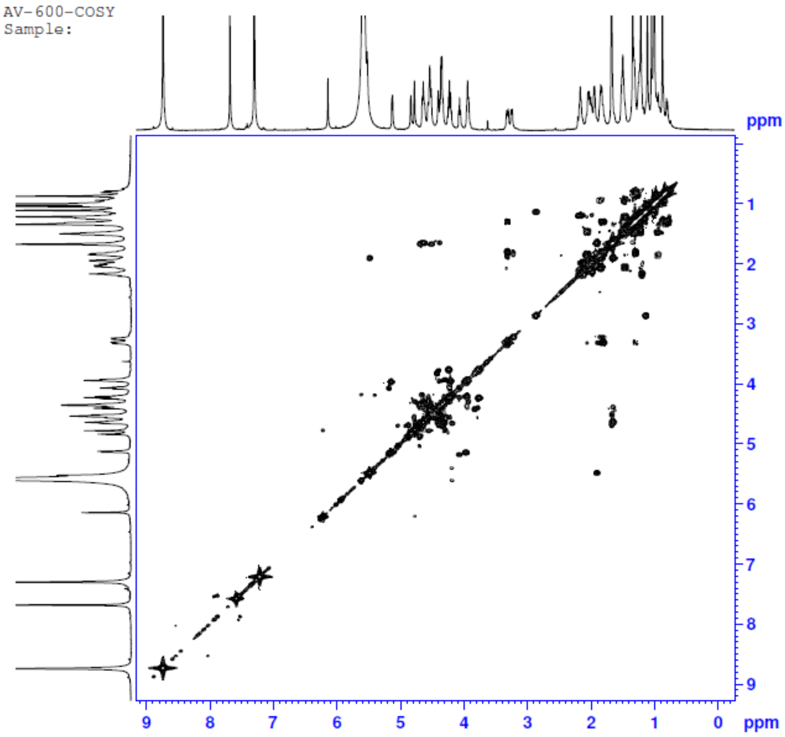

Figure S55. HSQC of Patrinia-glycoside B-II.

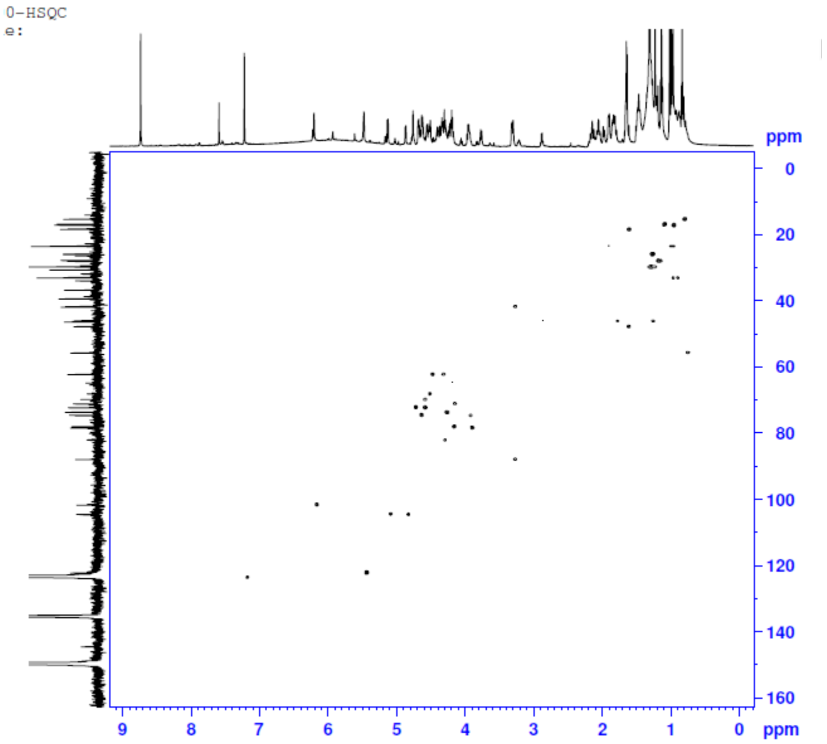

Figure S56. HMBC of Patrinia-glycoside B-II.

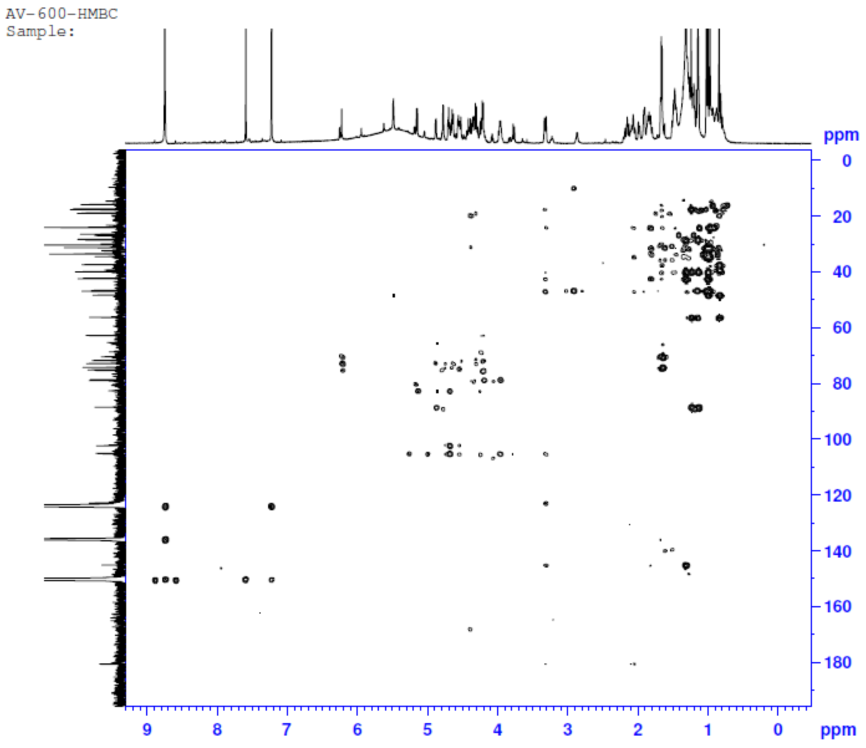

Figure S57. NOESY of Patrinia-glycoside B-II.

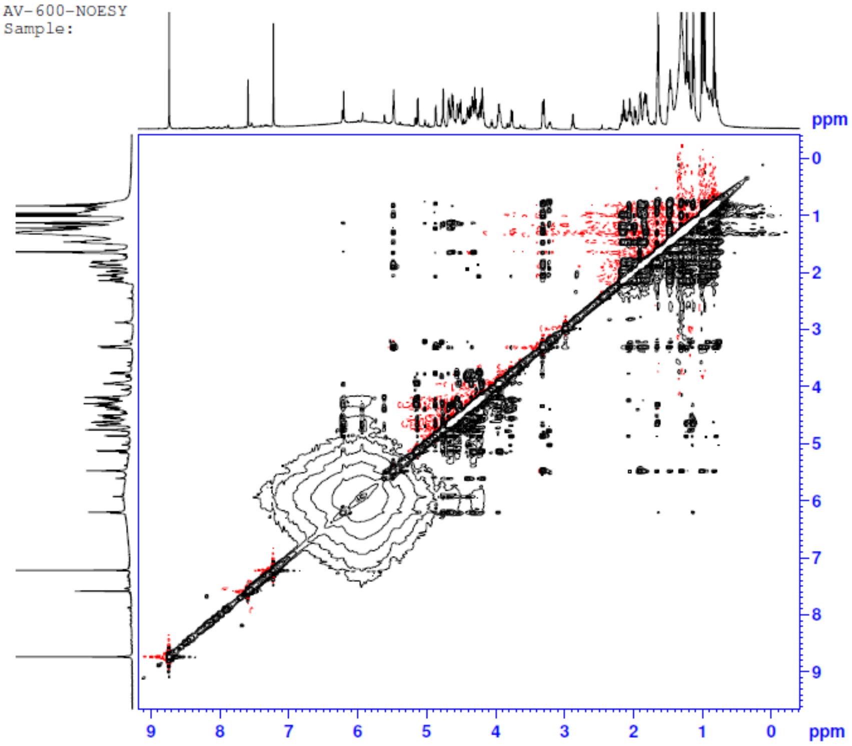

Supplement: Supplementary file 1 [file molecules-18-15193-s001.pdf]
